# Treatment options to support the elimination of hepatitis C: an open-label, factorial, randomised controlled non-inferiority trial

**DOI:** 10.1016/S0140-6736(25)00097-2

**Published:** 2025-05-08

**Authors:** Graham S Cooke, Hung Le Manh, Barnaby Flower, Leanne McCabe, Hang Vu Thi Kim, Thu Vo Thi, Thuan Dang Trong, Dung Nguyen Thanh, Phuong Le Thanh, Khoa Dao Bach, An Nguyen Thi Chau, Thach Pham Ngoc, Huong Vu Thi Thu, Bich Dang Thi, Tuyen Nguyen Kim, M Azim Ansari, Chau Le Ngoc, Quang Vo Minh, Phuong Nguyen Thi Ngoc, Thao Le Thi, Tran Nguyen Bao, Evelyne Kestelyn, Cherry Kingsley, Rogier Van Doorn, Motiur Rahman, Sarah L Pett, Guy E Thwaites, Eleanor Barnes, Jeremy N Day, Chau Nguyen Van Vinh, A Sarah Walker

**Affiliations:** NIHR BRC Imperial College NHS Trust, London, UK; Department of Infectious Disease, Imperial College London, London, UK; Hospital for Tropical Diseases, Ho Chi Minh City, Viet Nam; NIHR BRC Imperial College NHS Trust, London, UK; Oxford University Clinical Research Unit (OUCRU), Ho Chi Minh City, Viet Nam; Department of Infectious Disease, Imperial College London, London, UK; MRC Clinical Trial Unit at UCL, University College London, London, UK; Oxford University Clinical Research Unit (OUCRU), Ho Chi Minh City, Viet Nam; Oxford University Clinical Research Unit (OUCRU), Ho Chi Minh City, Viet Nam; Oxford University Clinical Research Unit (OUCRU), Ho Chi Minh City, Viet Nam; Hospital for Tropical Diseases, Ho Chi Minh City, Viet Nam; Hospital for Tropical Diseases, Ho Chi Minh City, Viet Nam; Hospital for Tropical Diseases, Ho Chi Minh City, Viet Nam; Oxford University Clinical Research Unit (OUCRU), Ho Chi Minh City, Viet Nam; National Hospital of Tropical Diseases, Hanoi, Viet Nam; National Hospital of Tropical Diseases, Hanoi, Viet Nam; National Hospital of Tropical Diseases, Hanoi, Viet Nam; Oxford University Clinical Research Unit (OUCRU), Hanoi, Viet Nam; Oxford University Clinical Research Unit (OUCRU), Ho Chi Minh City, Viet Nam; Nuffield Department of Medicine, University of Oxford, Oxford, UK; Oxford University Clinical Research Unit (OUCRU), Ho Chi Minh City, Viet Nam; Hospital for Tropical Diseases, Ho Chi Minh City, Viet Nam; Oxford University Clinical Research Unit (OUCRU), Ho Chi Minh City, Viet Nam; Oxford University Clinical Research Unit (OUCRU), Ho Chi Minh City, Viet Nam; Oxford University Clinical Research Unit (OUCRU), Ho Chi Minh City, Viet Nam; Oxford University Clinical Research Unit (OUCRU), Ho Chi Minh City, Viet Nam; Nuffield Department of Medicine, University of Oxford, Oxford, UK; NIHR BRC Imperial College NHS Trust, London, UK; Department of Infectious Disease, Imperial College London, London, UK; Oxford University Clinical Research Unit (OUCRU), Hanoi, Viet Nam; Nuffield Department of Medicine, University of Oxford, Oxford, UK; Oxford University Clinical Research Unit (OUCRU), Ho Chi Minh City, Viet Nam; MRC Clinical Trial Unit at UCL, University College London, London, UK; Hospital for Tropical Diseases, Ho Chi Minh City, Viet Nam; Oxford University Clinical Research Unit (OUCRU), Ho Chi Minh City, Viet Nam; Nuffield Department of Medicine, University of Oxford, Oxford, UK; Nuffield Department of Medicine, University of Oxford, Oxford, UK; NIHR BRC Oxford University NHS Trust, Oxford, UK; Hospital for Tropical Diseases, Ho Chi Minh City, Viet Nam; Oxford University Clinical Research Unit (OUCRU), Ho Chi Minh City, Viet Nam; Department of Clinical Microbiology and Infection, Royal Devon University Healthcare, Exeter, UK; Hospital for Tropical Diseases, Ho Chi Minh City, Viet Nam; MRC Clinical Trial Unit at UCL, University College London, London, UK

## Abstract

**Background:**

WHO recommends treating hepatitis C infection with one of three antiviral combinations for 8–12 weeks. No randomised trials have compared these regimens, and high cure rates might be achievable with shorter durations of therapy. We aimed to compare sofosbuvir–daclatasvir with sofosbuvir–velpatasvir, and to evaluate potential novel treatment strategies.

**Methods:**

We conducted a multi-arm, open-label, randomised controlled non-inferiority trial in two public hospitals in Viet Nam. Adults (aged ≥18 years) with chronic hepatitis C infection and mild-to-moderate liver fibrosis were eligible. Recruitment was stratified by centre and viral genotype (1–5 *vs* 6) with 1:1 random allocation to an oral fixed-dose combination of sofosbuvir 400 mg plus daclatasvir 60 mg (sofosbuvir–daclatasvir) or sofosbuvir 400 mg plus velpatasvir 100 mg (sofosbuvir–velpatasvir). Participants were simultaneously factorially randomly assigned to one of four treatment strategies: 12 weeks’ standard of care (SOC); 4 weeks’ therapy with four weekly PEGylated interferon alfa-2a subcutaneous injections; induction and maintenance therapy with 2 weeks’ standard therapy followed by 10 weeks’ therapy 5 days a week; and response-guided therapy (RGT) for 4, 8, or 12 weeks determined by viral load on day 7. The primary outcome was sustained virological response (SVR) 12 weeks after treatment completion, analysed in all evaluable participants regardless of actual treatment received. We chose a 5% non-inferiority margin for the drug comparison, and a 10% non-inferiority margin for the treatment strategy comparisons. Safety was assessed in all randomised participants. This trial is registered with ISRCTN, 61522291, and is completed.

**Findings:**

Between June 19, 2020, and May 10, 2023, 624 participants were randomised (470 [75%] were male and 154 [25%] were female). 296 (47%) had genotype 6 and 328 (53%) had genotypes 1–5. The primary outcome was assessable in 609 (98%) participants. SVR occurred in 294 (97%) of 302 participants in the sofosbuvir–daclatasvir group and 292 (95%) of 307 participants in the sofosbuvir–velpatasvir group (risk difference 2·2%, 90% credible interval [CrI] –0·2 to 4·8, within the 5% non-inferiority margin; 93% probability that sofosbuvir–daclatasvir is superior to sofosbuvir–velpatasvir). SVR occurred in 148 (99%) of 150 in the SOC group, 143 (94%) of 152 in the 4-week antiviral plus interferon group (–4·5%, 90% CrI –8·3 to –1·3), 151 (99%) of 152 in the induction–maintenance group (0·6%, –1·1 to 2·7), and 144 (93%) of 155 in the RGT group (–5·7%, –9·6 to –2·3); all risk differences were within the 10% non-inferiority margin. Serious adverse events were rare (11 [4%] of 313 participants in the sofosbuvir–velpatasvir group *vs* six [2%] of 311 in the sofosbuvir–daclatasvir group; risk difference –1·6% [95% CrI –4·2 to 0·8]) with no evidence of differences between regimens or strategies, but adverse reactions were very common in the 4-week antiviral plus interferon group compared with the other treatment strategies (risk difference *vs* SOC group, 66·8% [59·2 to 74·0]; p<0·0001).

**Interpretation:**

Sofosbuvir–daclatasvir was non-inferior to sofosbuvir–velpatasvir. High efficacy was seen with novel strategies, which might help to inform approaches to treatment for harder-to-reach populations.

**Funding:**

Wellcome Trust.

## Introduction

The development of direct-acting antivirals for hepatitis C virus (HCV) infection has transformed individual treatment and underpins ambitious WHO targets for global elimination of viral hepatitis as a public health threat by 2030.^1^ Of three WHO-recommended pan-genotypic first-line treatment options, two are preferred by lower-income and lower-middle-income countries and are available from generic manufacturers: sofosbuvir–daclatasvir and sofosbuvir–velpatasvir.^2^ No randomised trials have compared these treatment combinations and, in the absence of head-to-head comparisons and with high variability in generic pricing, countries differ in their choice of first-line treatment.^2^

In addition, the prevalence of viral genotypes and subtypes varies by region.^3^ Some lineages that are poorly represented in high-income countries and licensing trials have been shown to have poor outcomes as treatment access has expanded,^4^ raising questions about the generalisability of cure rates with pan-genotypic antivirals. More evidence is required from rarer genotypes to inform large-scale treatment roll-out.

For many high-burden countries, the current priority is scaling up simplified approaches to treatment.^5,6^ However, in the increasing number of settings where most of those engaged in care have been successfully treated, greater emphasis is needed on treatment strategies tailored to the smaller number of individuals for whom access to treatment and completion of standard 8–12 week therapy is challenging.^7^ A range of effective options that could be tailored to individual care based on patient, provider, and health system preferences could be invaluable, particularly strategies suitable for supervised therapy and which might reduce treatment costs. Such approaches have been widely explored in tuberculosis,^8^ but have very scarce evidence in HCV.

In this Article, we describe the first randomised controlled trial of WHO-approved first-line treatments for HCV, designed to assess non-inferiority of sofosbuvir–daclatasvir versus sofosbuvir–velpatasvir, and to evaluate potential novel treatment strategies that could expand access to treatment using an efficient platform factorial design.^9^

## Methods

### Study design

This was an open-label, multicentre, factorial randomised non-inferiority trial in two public hospitals in Viet Nam (Hospital for Tropical Diseases, Ho Chi Minh City, and National Hospital of Tropical Disease, Hanoi).^9^ Ethical approval was obtained from local hospital ethics committees at the Hospital for Tropical Diseases and the National Hospital of Tropical Disease; the Ministry of Health, Viet Nam; Imperial College London, UK; and the University of Oxford, UK (ref 110-0319/35CN). This trial is registered with ISRCTN, 61522291.

### Participants

Participants were adults aged 18 years and older with chronic HCV infection, mild to moderate liver disease (FibroScan result of ≤9 kPa, or Fibrosis-4 [FIB-4] Index for Liver Fibrosis score of ≤1·45 within the 180 days before enrolment), and with a known HCV genotype. HIV-positive participants were eligible if they were on stable antiretroviral therapy, but were switched to integrase-based therapy (if not already taking) for the duration of the trial to avoid drug–drug interactions. HBsAg-positive participants not on active therapy for hepatitis B virus infection were eligible but were treated with tenofovir disproxil fumarate within the trial (appendix p 4). Full eligibility criteria are listed in the appendix (pp 3–4). Participants were recruited by the trials team from routine clinics and provided written informed consent before random assignment.

### Randomisation and masking

Randomisation was stratified by centre and genotype 6 versus non-genotype 6 (ie, genotype 1–5). Randomisation used a computer-generated sequential list with variably sized permuted blocks prepared by the trial statistician (LM) and incorporated securely into the online trial database. The list was concealed until allocation of eligible participants by local trial centre staff with random assignment performed on treatment day 0.

**Figure 1:**
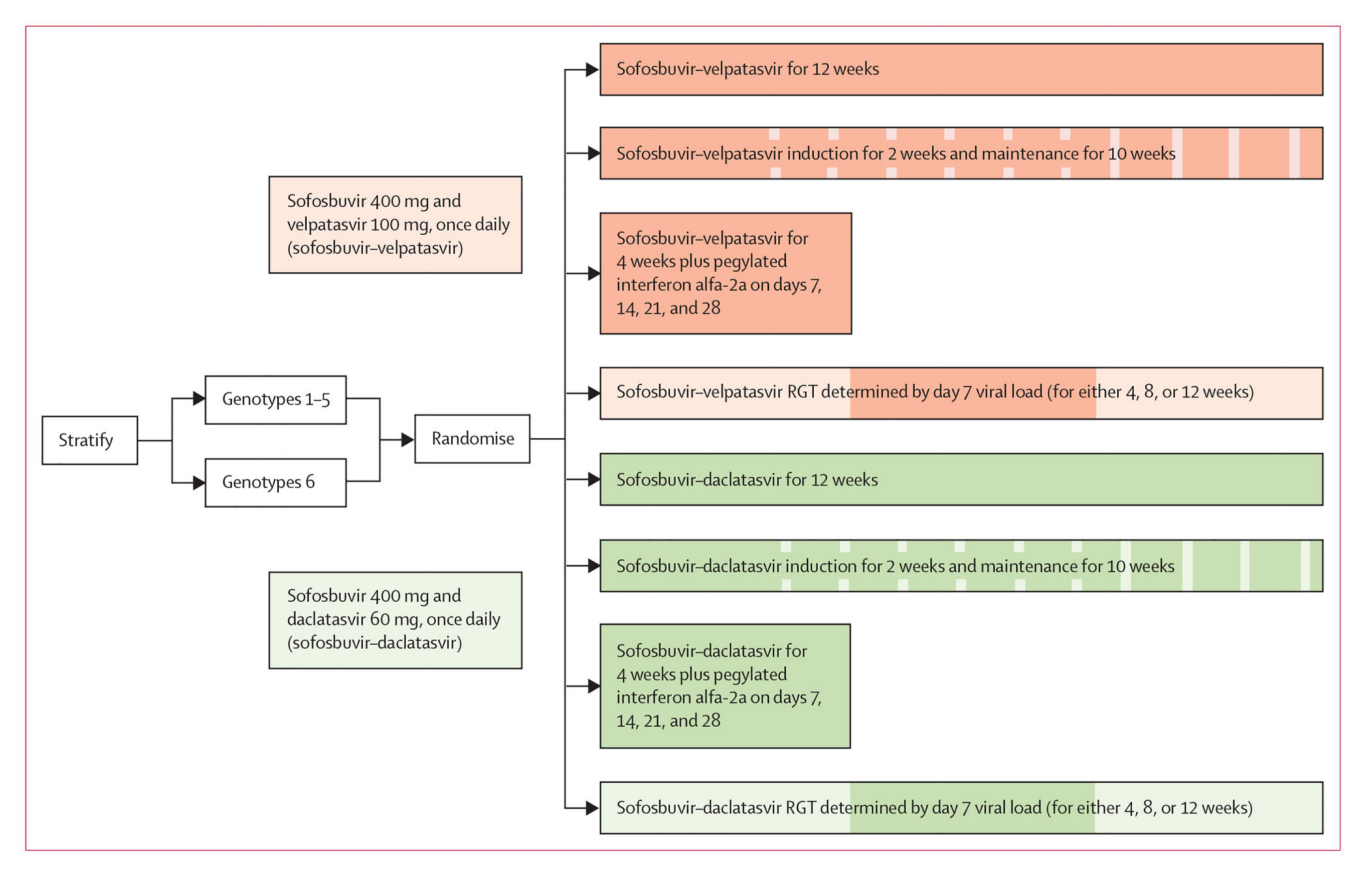
Trial design Random assignment was stratified by centre and viral genotype. RGT=response guided therapy.

Individuals were randomly assigned 1:1 to the sofosbuvir–daclatasvir group or the sofosbuvir–velpatasvir group (figure 1). Patients were simultaneously factorially randomly assigned 1:1:1:1 to one of four treatment strategies: (1) standard of care (SOC group); (2) 4 weeks’ therapy with four additional weekly PEGylated interferon alfa-2a subcutaneous injections (4-week antiviral plus interferon group); (3) induction with 2 weeks’ daily therapy followed by 10 weeks’ maintenance therapy 5 days per week with weekends off (induction–maintenance group); and (4) response-guided therapy (RGT) with duration determined by a single quantitative viral load on day 7 (RGT group). The trial was open-label: no placebo was provided due to the complexity of ensuring that participants took the right pills on the right days. Masking of trial staff from randomised assignment was not possible, but efficacy endpoints were based on HCV viral loads assessed in the laboratory by masked individuals.

In the original protocol there was an additional factorial randomisation to adjunctive ribavirin or no ribavirin. Only 22 participants were allocated ribavirin before this group was stopped on Jan 26, 2021, on advice of the trial steering committee in light of recruitment challenges during the COVID-19 pandemic, and emerging evidence of a lack of efficacy of adjunctive ribavirin.^10^

### Procedures

Participants in the sofosbuvir–daclatasvir group received sofosbuvir 400 mg and daclatasvir 60 mg as an oral fixed-dose combination, and those in the sofosbuvir–velpatasvir group received sofosbuvir 400 mg and velpatasvir 100 mg as an oral fixed-dose combination. In the SOC group, participants were instructed to take treatment daily for 12 weeks. In the 4-week antiviral plus interferon group, participants received 4 weeks’ standard daily therapy and four additional weekly subcutaneous injections of 180 μg PEGylated interferon alfa-2a on days 7, 14, 21, and 28. In the induction–maintenance group, after 2 weeks of standard daily therapy, participants were instructed to take pills on weekdays (ie, Monday to Friday) until day 56 (and were provided with sufficient pills to last until day 56, excluding weekends); on day 56 they received the same instructions and a pill supply to last until day 84. In the RGT group, participants with a viral load less than the lower level of quantification at day 7 received a total of 4 weeks of therapy, those with a viral load of more than the lower level of quantification and less than 250 IU/mL received 8 weeks, and the remainder received 12 weeks. All trial drugs were purchased at market prices through generic suppliers (direct-acting antivirals from Mylan, Mumbai, India; PEGylated interferon alfa-2a from Zuillig Pharma, Ho Chi Minh City, Viet Nam).

Baseline characteristics were collected by the trial team through clinical assessment with participants at the baseline visit. Data on sex (male or female) were collected by self-report; we did not collect data on ethnicity. Viral load was quantified using the COBAS AmpliPrep/COBAS TaqMan HCV Quantitative Test version 2.0 (Roche Diagnostics, Basel, Switzerland; lower level of quantification 15 IU/mL) or m2000 real-time HCV assay (Abbott, Illinois, USA; lower level of quantification 12 IU/mL).

Timing of assessments differed slightly according to the varying first-line treatment durations in each treatment strategy (appendix p 7). At each assessment conducted while the participant was taking direct-acting antivirals, adherence was assessed using self-report and pill counts. At each assessment on or off direct-acting antivirals, adverse events that were serious, clinical and grade 3 or 4 assessed using the Common Terminology Criteria for Adverse Events version 5.0 grading scale, potentially related to HCV treatment, or that led to a change in HCV treatment were actively solicited. Participants could also return to the clinic at any time for unscheduled assessments if adverse events occurred.

Following completion of first-line treatment, all participants were scheduled for three visits at 28-day intervals until the primary endpoint 12 weeks after end of treatment. In response to societal lockdowns to tackle the COVID-19 pandemic, recruitment was paused at one or both sites between May 24, 2021, and Feb 12, 2022. During this period, follow-up visits after the end of treatment were conducted remotely via telephone, with confirmation of sustained virological response (SVR) at 12 weeks after the end of treatment postponed beyond 12 weeks in some participants, once lockdowns had lifted.

Individuals not achieving SVR 12 weeks after finishing first-line treatment were re-treated with 12 weeks of the alternative treatment combination they were not randomly assigned to receive first-line, plus ribavirin if not contraindicated.

### Outcomes

The primary endpoint was SVR, defined as plasma HCV RNA less than the lower level of quantification 12 weeks after the end of treatment without previous treatment failure. Failure of first-line treatment was carefully defined to incorporate individuals with fully suppressed HCV RNA (ie, less than the lower level of quantification) on therapy with late virological rebound and those without full suppression of the HCV viral load. In both cases, two consecutive viral loads more than the lower level of quantification, taken at least 1 week apart after the end of treatment, were required to confirm treatment failure, with the second required to be more than 2000 IU/mL.

Secondary outcomes were SVR 12 weeks after combined first-line or re-treatment; lack of initial virological response (<1 log_10_ HCV RNA decrease from baseline); serious adverse events, grade 3 or 4 clinical adverse events, adverse events of any grade leading to change in treatment, and adverse events of any grade that were classified by the investigator as possibly, probably, or definitely related to trial treatment; and emergence of resistance-associated substitutions in individuals not achieving SVR (assays ongoing; results to be reported separately).

### Statistical analysis

Sample size was based on two components. First, given the novel strategies being tested, each group was monitored as a single group intervention trial, comparing SVR rates to a target of 90%. 39 participants in each genotype strata (two strata) and randomly assigned group (eight groups consisting of two regimens by four strategies) were required to detect an unacceptably low cure rate of 70% with 90% power, a one-sided α of 0·05, and 5% loss to follow-up, resulting in a total of 624 required participants. Second, random assignment of 624 participants provided 88% power to demonstrate non-inferiority between treatment combinations based on a non-inferiority margin of 5%, and 99% power to demonstrate non-inferiority between treatment strategies based on a non-inferiority margin of 10%, assuming 95% SVR, a one-sided α of 0·05, and 5% loss to follow-up. A 5% non-inferiority margin was chosen for the drug comparison since only a small difference in efficacy would potentially be enough to favour one of the two options in practice, as they are intended for widespread use at scale. A slightly wider, more traditional 10% non-inferiority margin was set for the treatment strategy comparisons given the other advantages they would provide in treating hard-to-reach populations in whom adherence and standard 12-week regimens are more challenging. The data monitoring committee held three meetings to review data; they did not stop treatment in any of the arms (appendix p 4).

The primary efficacy analysis was done in the modified intention-to-treat population, which included all participants in whom the primary endpoint could be evaluated regardless of actual treatment received. The primary safety analysis was done in all randomised patients. A secondary per-protocol efficacy analysis restricted to participants taking 90–110% of prescribed treatment would have been performed if more than 10% of participants had been excluded from it. Primary comparisons between randomised groups were analysed using Bayesian methods with uninformative priors, calculating marginal risk differences and 90% credible intervals (CrIs) after logistic regression, reflecting the non-inferiority design, since it is implausible that giving less treatment could lead to better outcomes. Secondary analyses used sceptical and enthusiastic priors (appendix p 39), 95% CrIs, and frequentist methods. We also calculated probabilities of difference from these Bayesian models; P denotes a one-sided probability that the difference versus a reference is less than zero following the non-inferiority design. p denotes a two-sided frequentist p value.

All analyses were adjusted for the other randomisation and stratification factors (genotype 6 *vs* genotypes 1–5 and centre).^11^ Heterogeneity across subgroups in the effects of the treatment combination and treatment strategy on the primary endpoint was investigated using interaction tests in frequentist logistic models, for ten prespecified and five additional exploratory factors (appendix p 7). Logistic models were penalised when cure rates were 100% in one or more subgroups and models without penalisation would not converge. The proportion of participants with undetectable HCV viral load at each visit was analysed using χ^2^ or exact tests and binomial generalised estimating equations with an independent correlation structure, using global tests of difference to compare randomised drug and treatment strategy groups. Absolute HCV viral load (log_10_ scale) at day 7 or 14, as scheduled depending on treatment strategy, was analysed using interval regression adjusting for baseline log_10_(HCV viral load) with the left interval set to minus infinity and the right interval set to log_10_ (lower level of quantification) for those with undetectable viral load, and the upper and lower limits of the interval set to set to log_10_(observed value) for those with detectable viral load. Bayesian analyses were done with R version 4.1.3, all other analyses were done using Stata version 18.0 (appendix pp 5–6).

### Role of the funding source

The funder of the study had no role in the study design, data collection, data analysis, data interpretation, or writing of the report.

## Results

**Figure 2:**
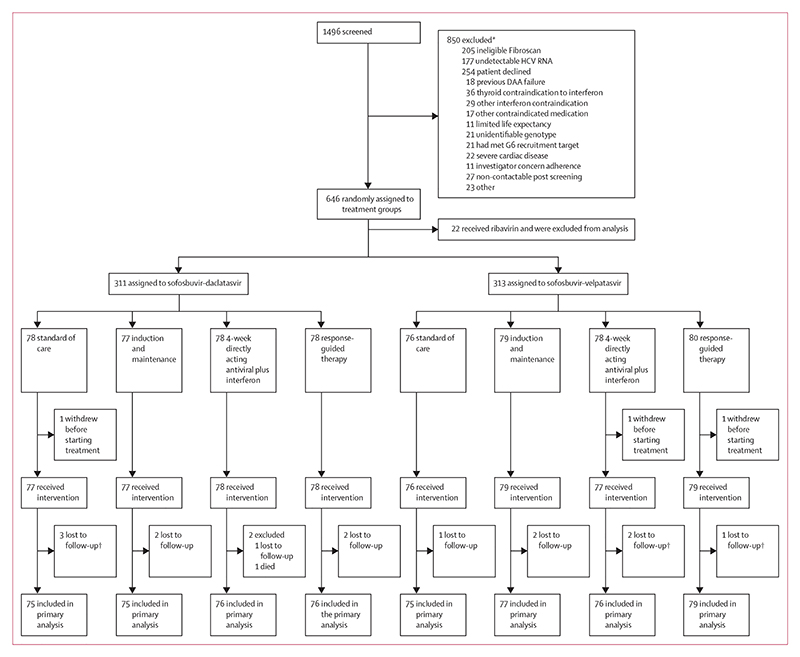
CONSORT diagram Three participants withdrew immediately after random assignment without starting treatment so could not be evaluated for the primary endpoint. DAA=direct-acting antiviral. HCV=hepatitis C virus. SOC=standard of care. *Individuals might have had more than one reason for exclusion. †One patient lost to follow-up in this group was still eligible for inclusion in the primary analysis as they had provided data for viral load at 12 weeks after end of treatment (appendix p 5).

Between June 19, 2020, and May 10, 2023, 646 participants were randomly assigned to treatment groups (figure 2). 22 individuals were factorially randomised to adjunctive treatment with ribavirin and subsequently excluded from all analyses, resulting in a final sample of 624 individuals. 78 people were assigned to receive sofosbuvir–daclatasvir and 76 to sofosbuvir–velpatasvir in the SOC group; 77 were assigned to receive sofosbuvir–daclatasvir and 79 to sofosbuvir–velpatasvir in the induction and maintenance group; 78 were assigned to receive sofosbuvir–daclatasvir and 78 to sofosbuvir–velpatasvir in the 4-week antiviral plus interferon group; and 78 were assigned to receive sofosbuvir–daclatasvir and 80 to sofosbuvir–velpatasvir in the RGT group. Baseline characteristics were broadly balanced between groups (table 1; appendix p 19). Median age was 42 years (IQR 37–51), 154 (25%) patients were female, 84 (13%) were HIV positive (and receiving treatment, in line with the inclusion criteria), and 213 (34%) had a history of illicit substance use. 296 (47%) were infected with genotype 6. Of non-genotype 6 infections, the most common was genotype 1, followed by genotype 2 and genotype 3 (table 1).

Three (1%) participants withdrew immediately after random assignment and never started treatment (figure 2). In the remaining 621 participants, the number of days for which a first-line direct-acting antiviral was taken varied substantially across arms, with a mean of 28·0 days (SD 0·4) in the 4-week antiviral plus interferon group, 52·9 days (17·5) in the RGT group, 62·9 days (3·1) in the induction–maintenance group, and 83·4 (2·6) days in the SOC group (appendix p 21). In the RGT group, 40 (25%) of the 157 individuals starting randomised treatment received 4 weeks of direct-acting antivirals, 94 (60%) received 8 weeks, and 23 (15%) received 12 weeks (appendix p 22). 584 (94%) of 624 completed first-line therapy as allocated, and 607 (97%) received 90–110% of allocated first-line therapy (per-protocol population) such that a separate analysis of this population was not performed as per the statistical analysis plan. In total, 16 (3%) participants were lost to follow-up (nine [1%] before end of first-line treatment, six [1%] after the end of treatment, and one [<1%] during re-treatment; appendix p 26), of whom five (1%) formally withdrew consent and one (<1%) died after completing first-line treatment (cause unknown; last previous HCV viral load undetectable). Only 46 (7%) individuals missed any first-line visit and 82 (13%) reported missing any allocated direct-acting antiviral doses (appendix p 22). Missed doses occurred significantly more often in the SOC group (35 [23%] in the SOC group *vs* 18 [11%] in the RGT group, ten [6%] in the 4-week antiviral plus interferon group, and 19 [12%] in the induction–maintenance group; p<0·0001; appendix p 22), particularly after day 28. Nine (6%) of 156 participants in the 4-week antiviral plus interferon group reported missing any interferon doses, and 32 (21%) of 156 participants in the induction–maintenance group reported taking weekend doses.

Primary outcome data were available for 609 (98%) participants. Overall, SVR 12 weeks after the end of first-line treatment was achieved in 294 (97%) of 302 in the sofosbuvir–daclatasvir group versus 292 (95%) of 307 participants in the sofosbuvir–velpatasvir group (risk difference 2·2% [90% CrI –0·2 to 4·8], which is within the 5% non-inferiority margin, P=0·069; table 2), with a 93% probability of higher efficacy from sofosbuvir–daclatasvir versus sofosbuvir–velpatasvir (appendix p 23).

Overall, SVR 12 weeks after the end of first-line treatment was achieved for 148 (99%) of 150 participants in the SOC group, 151 (99%) of 152 in the induction–maintenance group (risk difference 0·6% [90% CrI –1·1 to 2·7]), 143 (94%) of 152 in the 4-week antiviral plus interferon group (–4·5%, –8·3 to –1·3), and 144 (93%) of 155 in the RGT group (–5·7%, –9·6 to –2·3), all within the 10% non-inferiority margin. There was a 26% probability that induction–maintenance was inferior to SOC, a 99% probability that 4-week antiviral plus interferon was inferior to SOC, and a 100% probability that RGT was inferior to SOC (table 2). There was no evidence of heterogeneity in the effect of the two factorial randomisations (penalised logistic regression heterogeneity p=0·79), nor in the effect of treatment regimen or strategy by nine other prespecified and five additional exploratory factors (appendix pp 8–13, 25–28). In particular, there was no evidence that *IFNL4* genotype modified the effect of the regimen (heterogeneity p=0·80; appendix p 8) or treatment strategy, including for the comparison between the 4-week antiviral plus interferon group versus the SOC group (risk difference –4·7% [95% CI –8·9 to 0·5] for the *IFNL4* CC genotype and –1·2% [–18·1 to 15·6] for the *IFNL4* CT or TT genotype; appendix p 8). Similarly, there was no evidence that baseline HCV viral load (heterogeneity as a continuous factor p=0·60; appendix pp 12–13) or degree of fibrosis (p=0·22) modified the effect of the regimen or the treatment strategy (heterogeneity p=0·27 and p=0·49, respectively). SVR for those in the RGT group receiving 4 weeks of therapy (who had a viral load less than the lower level of quantification at day 7) occurred in 31 (80%) of 39 participants (appendix p 29). All but four participants had an initial virological response (table 2).

First-line SVR occurred more often in participants with HCV genotype 3 (22 [100%] of 22) and genotype 6 (281 [98%] of 288) than in those with genotype 2 (43 [88%] of 49). In adjusted exploratory analyses, the first-line SVR rate tended to be higher in participants with HCV genotype 6 than in those with non-genotype 6 infection (risk difference 2·6%, 95% CrI –0·3 to 5·7, P=0·080, whether adjusting for human genetic polymorphisms in the *IFNL4* gene or not). However, this finding was due to a lower SVR rate in participants with genotype 2 compared with those with genotypes 1 and 3 combined (in fully adjusted models, risk difference –15·0% [95% CrI –30·4 to –3·8], P=0·0031) with no evidence of differences between participants with genotype 6 versus those with genotypes 1 and 3 combined (1·0%, –1·6 to 3·9, P=0·44; appendix p 30). There was weak evidence for a slightly higher response in participants with the *IFNL4* CT and TT genotypes compared with the *IFNL4* CC genotype as expected (in fully adjusted models 3·5%, –0·7 to 9·7, p=0·12), and of higher responses overall with sofosbuvir–daclatasvir compared with sofosbuvir–velpatasvir (2·7%, –0·2 to 5·6, P=0·065). There was no evidence of interaction between genotype and regimen (heterogeneity p=0·70).

All 23 SVR failures were in individuals who had an HCV viral load less than the lower level of quantification at end of treatment, 17 of which were undetectable (appendix pp 31–32). Most failures were detected 4 weeks after end of treatment (12 [52%] of 23) or 8 weeks after end of treatment (nine [39%]). 20 (87%) of the individuals with SVR failure had two consecutive HCV viral load measurements of less than the lower level of quantification that then rebounded. The remaining three individuals (13%) had at most one HCV viral load less than the lower level of quantification. The median HCV viral load was 308 000 IU/mL (IQR 47 098–8 120 000) at treatment failure. Four (44%) of nine treatment failures in the 4-week antiviral plus interferon group occurred in individuals who had missed one (n=3) or two (n=1) interferon doses (appendix p 32); all four of these individuals also received sofosbuvir–velpatasvir. Eight (35%) of the 23 overall treatment failures were in people who received only 4 weeks of direct-acting antiviral in the RGT group.

Re-treatment regimens were based on the treatment combination not used first, plus ribavirin if it was not contraindicated; sofosbuvir–daclatasvir and ribavirin were given to 15 individuals, sofosbuvir–velpatasvir and ribavirin to six, and sofosbuvir–velpatasvir to two, and were initiated a median of 4 weeks (IQR 2–8) after treatment failure. All but two participants, both initially randomly assigned to sofosbuvir–velpatasvir (one in the induction–maintenance group, and one in 4-week antiviral plus interferon group), achieved an SVR following re-treatment, leading to an overall cure rate for first-line plus re-treatment of more than 99% (607 of 609; table 2).

At day 7, HCV viral load was measured only in the RGT group; undetectable HCV viral load was obtained in 44 (27%) of 163 participants (appendix pp 8, 14). At day 14, HCV viral load was measured in all groups except the RGT group; undetectable HCV viral load was obtained in 277 (58%) of 474 individuals. There was no evidence of differences by regimen in the percentage of individuals with undetectable HCV viral load over time until 12 weeks after the end of treatment (global p=0·54; appendix p 18) or in the change in HCV viral load to day 7 or day 14 (p>0·19; appendix p 20). In contrast, a significantly higher percentage of participants in the 4-week antiviral plus interferon group had an undetectable HCV viral load at day 14 than in the SOC group (117 [74%] of 159 *vs* 89 [59%] of 152, p=0·0051; the only difference between the two groups in treatment received at this viral load measurement was the receipt of a single interferon dose on day 7; appendix p 15) and a significantly lower percentage of the induction–maintenance group had an undetectable HCV viral load at day 14 than in the SOC group (71 [44%] of 163; p=0·0078) despite the SOC and induction–maintenance groups receiving identical treatment up to this timepoint, with similar differences observed between these strategies in the change in HCV viral load to day 7 or 14 (appendix p 17).

Overall, serious adverse events were rare, occurring in 17 (3%) of 624 participants, as were grade 3 or 4 adverse events; there was no evidence of differences between treatment combinations or strategies (table 3; appendix pp 33–34). Four adverse events were judged by the investigator to be related to treatment (appendix p 34; all related to interferon, notably leucopenia and thrombocytopenia). One death occurred in the 4-week antiviral plus interferon group 2 weeks after completion of treatment; the cause was not established but was judged to be unrelated to treatment. Adverse reactions were uncommon in the SOC (five [3%] of 154), induction–maintenance (six [4%] of 156), and RGT groups (five [3%] of 159), but very common in the 4-week antiviral plus interferon group (109 [70%] of 156; appendix p 35). Most reactions were low grade fever or influenza-like illness or haematological abnormalities of relatively short duration (median 2 days) and they only led to a change in treatment in four (3%) of 156 participants in the 4-week antiviral plus interferon group (appendix pp 35–37). Low-grade haematological abnormalities were common at end of the 4-week treatment period in the antiviral plus interferon group (50 [34%] of 149 had neutropenia, 15 [10%] had leucopenia, and 16 [11%] had thrombocytopenia) but neutropenia had resolved in all but two, leucopenia had resolved in all but three, and thrombocytopenia in all 125 participants by 12 weeks after treatment (appendix p 38).

## Discussion

WHO hepatitis treatment guidelines currently recommend three pan-genotypic combinations: sofosbuvir–daclatasvir, sofosbuvir–daclatasvir, or glecaprevir–pibrentasvir, but glecaprevir–pibrentasvir is seldom available in lower-income and lower-middle-income countries where disease burden is highest, and most low-income countries prefer either sofosbuvir–daclatasvir or sofosbuvir–velpatasvir for first-line therapy. Despite receiving market authorisation in 2015, daclatasvir is no longer commercially available in high-income countries and is therefore not recommended as an option in major international treatment guidelines.^6,13^ However, sofosbuvir–daclatasvir is the most widely available HCV treatment globally with the most generic manufacturers worldwide. Sofosbuvir–daclatasvir can be procured through voluntary licences for as little as US$60 per course, although prices vary considerably between countries (with some as high as $963). In some countries (eg, Viet Nam), sofosbuvir–velpatasvir^2^ is the preferred first-line treatment, with prices varying from $100 to $4000 in different lower-income and lower-middle-income countries.

In this study, we found that sofosbuvir–daclatasvir was non-inferior to sofosbuvir–velpatasvir, and the high cure rates in both sofosbuvir–daclatasvir and sofosbuvir–velpatasvir standard-of-care 12-week treatment groups support the use of both of these treatment combinations. However, when comparing all those treated with sofosbuvir–daclatasvir versus sofosbuvir–velpatasvir, sofosbuvir–daclatasvir had a 93% probability of having a higher efficacy. These findings support the role of sofosbuvir–daclatasvir as a preferred first-line therapy, although further head-to-head studies in different settings would be helpful.

Of the different novel treatment strategies investigated through the factorial design in this study, the highest cure rates were in the induction–maintenance therapy group (99%), with a very similar result in the SOC group (99%), despite there being fewer doses in the induction–maintenance group (64 doses) than in the SOC group (84 doses). These data provide some evidence that improves our understanding of the effect of reduced adherence, and the findings support current guidance on managing patients with non-adherence.^6^ An induction and maintenance approach will not be widely applicable but might be helpful for the smaller number of individuals requiring directly supervised therapy, who cannot be supervised every day, as with some current tuberculosis regimens. In some settings, such a treatment strategy could be a more cost-effective treatment. Further work is required to explore whether less frequent dosing, such as three times a week, perhaps with higher individual doses, is as efficacious as standard of care, to provide even simpler options suitable for supervised community therapy.

Interferon-free options are now the preferred option for HCV treatment due to the well documented challenges of unacceptable toxicity with long courses of interferon (ie, 24–48 weeks).^14^ However, there remains some interest in the potential role of interferon in allowing ultrashort treatment for which side-effects due to interferon would be limited to a short time period,^15^ particularly for patients in whom standard 8–12 week courses of direct-acting antivirals remain challenging to complete. Furthermore, interferon remains in clinical use in many countries (eg, for the treatment of hepatitis B), supporting the potential value of exploring such ultrashort approaches. One small trial^15^ found high cure rates with 4 weeks of direct-acting antiviral therapy with ribavirin plus interferon; however, the trial population was limited to patients with low viral loads before treatment. In this study, we found that adding four doses of PEGylated interferon alfa-2a to 4 weeks of direct-acting antiviral therapy achieved high cure rates, and there was no evidence that the treatment outcome depended on baseline viral load or host *IFLN4* genotype. Furthermore, there was some evidence that the proportion of individuals with undetectable HCV viral load at day 14 in the 4-week antiviral plus interferon group was greater than in the SOC group, although the only difference in treatment received between these groups at this timepoint was the single interferon dose at day 7. Of note, four of nine individuals who had treatment failure in the 4-week antiviral plus interferon group reported missing interferon doses, highlighting the importance of interferon with short direct-acting antiviral therapy— particularly as eight of 11 individuals who had treatment failure in the RGT group had only received 4 weeks of direct-acting antivirals. However, as expected, interferon-related side-effects were common, but most were mild and short-lived. Although only likely to be suitable for a small number of patients, this treatment strategy could be an option when only a short window for therapy exists or when supervised therapy is being considered. Of note, 65 (8%) of 850 participants were excluded at screening due to potential risks of complications from interferon and, therefore, the treatment can only be considered an option for a selected proportion of patients.

The RGT group was able to achieve a high SVR with treatment duration determined by a single quantitative viral load at day 7, albeit at an additional cost and inconvenience. Although SVR in those receiving 4 weeks of therapy in the RGT group was higher than that seen with 4 weeks of therapy in previous studies,^16,17^ the overall SVR rate was only 93%. As such, this treatment strategy is less likely to appeal in practice than other strategies and would benefit from further evaluation—eg, by extending the treatment duration beyond 4 weeks for those with an undetectable viral load at day 7. However, the high SVR with 8 weeks of therapy supports the findings of some previous studies showing effectiveness of 8 weeks of direct-acting antivirals in mild liver disease.^18–20^

HCV is one of the most genetically diverse human viruses and the most diverse genotype is genotype 6, with related concerns about the potential for clinical resistance.^21,22^ Despite this reality, cure rates were higher in participants with HCV genotype 6 than those with non-genotype 6 with no evidence of heterogeneity in effects of sofosbuvir–daclatasvir and sofosbuvir–velpatasvir by genotype. Of note, SVR rates were unexpectedly lower in participants with genotype 2 than in those with other genotypes. This finding requires more investigation; the sample was small, so we cannot exclude this finding as being due to chance.

Limitations of this study include the fact that the trial only included individuals with mild to moderate liver disease; cure rates in those with more advanced fibrosis or cirrhosis will be lower and further work is needed to establish the role of shorter-course treatment in such individuals. We did not record active substance misuse during the trial, only history of illicit substance use at any time (current or past) due to concerns about the willingness of participants to disclose this information, so we were not able to assess the effect of active substance misuse on response. Masking of trial staff and participants was not possible, but efficacy endpoints were based on HCV viral loads assessed in the laboratory by individuals masked to randomised allocations. In-depth full length HCV deep sequencing is ongoing, so at present we cannot exclude the possibility that a small number of the 23 treatment failures could in fact be re-infections; however, as SVR was assessed only 12 weeks after end of treatment and in Viet Nam many chronic HCV infections are the consequence of historical drug use, re-infection was unlikely. In addition, cure rates in the SOC and induction–maintenance groups were very high and there is no reason to think re-infection rates would differ between groups. The primary outcome was intended to reflect the intention-to-treat effect of providing direct-acting antivirals, which would include re-infections in those with ongoing risk behaviours. If a small number of apparent treatment failures did in fact represent re-infections, it would suggest the true efficacy of the strategies tested would be higher than observed.

Both host and viral genetic variation might limit generalisability. Although the trial was open to all patients, it included very few participants with genotypes 3, 4, or 5 and therefore regimens and strategies cannot be assumed to have similar absolute efficacy in settings with different genotype distributions, which include the potential for differing genotype 6 variants in other populations in Asia (eg, in China). Despite concerns about naturally occurring resistance, genotype subtypes previously associated with NS5A resistance (6d, 6f, and 6r) were not found in this study. The Vietnamese population has a high background prevalence of the *IFNL4* allele associated with high interferon efficacy;^23^ further work is needed to establish the relevance of this prevalence to the outcome and applicability in other populations. Finally, these findings were obtained in a setting with high adherence and follow-up. Although high levels of adherence are not unusual in trial settings, adherence might have been increased as a result of the intensive follow-up schedule in this trial. SVR rates might be lower in real-world settings, although differences between groups should generalise.

When treatment costs are proportional to the number of tablets used, the induction and maintenance strategy would be cost saving, although this is not the primary intent of the strategy. Given their lower drug use but other additional costs beyond standard of care, the 4-week antiviral plus interferon and RGT strategies will be cost-effective in some settings, although the prices of both diagnostics and drugs vary greatly and will need to be assessed locally.

The strategic approaches tested here were all designed to have the potential to achieve high cure rates while providing a range of options for treatment. These treatments should not be considered as alternatives to each other, but rather as options that might be suitable for individual patients based on their preferences, those of their provider, the system they are cared for within, and affordability, to achieve the ultimate goal of global HCV elimination.

## Supplementary Material

supplementary file

## Figures and Tables

**Table 1:** Baseline characteristics by treatment combination and treatment strategy

	Treatment combination			Treatment strategy			
	Sofosbuvir–velpatasvir (n=313)	Sofosbuvir–daclatasvir (n=311)		Standard of care (n=154)	Induction and maintenance (n=156)	4-week antiviral plus interferon (n=156)	Response-guided therapy (n=158)
Age, years	42 (37–50)	42 (37–51)		43 (37–50)	42 (36–49)	43 (39–52)	42 (36–53)
Sex							
Female	80 (26%)	74 (24%)		40 (26%)	36 (23%)	38 (24%)	40 (25%)
Male	233 (74%)	237 (76%)		114 (74%)	120 (77%)	118 (76%)	118 (75%)
BMI, kg/m^2^	22·0 (20·3–24·5)	22·3 (20·3–24·4)		21·9 (20·3–24·9)	22·4 (20·4–24·5)	22·5 (20·7–24·6)	21·9 (19·8–24·0)
HCV viral load, log_10_ lU/mL	6·1 (1·1)	6·1 (1·0)		6·0 (1·0)	6·2 (1·1)	6·1 (1·0)	6·1 (1·1)
HCV genotype							
1	131 (42%)	126 (41%)		61 (40%)	62 (40%)	66 (42%)	68 (43%)
2	21 (7%)	28 (9%)		11 (7%)	14 (9%)	13 (8%)	11 (7%)
3	13 (4%)	9 (3%)		8 (5%)	6 (4%)	4 (3%)	4 (3%)
6	148 (47%)	148 (48%)		74 (48%)	74 (47%)	73 (47%)	75 (48%)
Fibroscan result, kPa	6·2 (5·1–7·4)	6·1 (5·0–7·1)		6·2 (5·3–7·2)	6·2 (5·0–7·4)	6·0 (5·0–7·0)	6·1 (4·9–7·6)
Missing data	4	5		6	0	2	1
HIV positive	41 (13%)	42 (14%)		21 (14%)	22 (14%)	18 (12%)	22 (14%)
HBsAg positive	16/312 (5%)	23/311 (7%)		8/154 (5%)	9/156 (6%)	14/152 (9%)	8/157 (5%)
*IFNL4* genotype							
CC	270/312 (87%)	263/310 (85%)		137/153 (90%)	128/156 (82%)	132/155 (85%)	136/158 (86%)
CT	17/312 (5%)	22/310 (7%)		6/153 (4%)	11/156 (7%)	13/155 (8%)	9/158 (6%)
TT	25/312 (8%)	25/310 (8%)		10/153 (7%)	17/156 (11%)	10/155 (6%)	13/158 (8%)

Data are median (IQR), n (%), or n/N (%) when the denominator differs from that in the column heading. All standardised mean differences (data not shown) are <0·2 suggesting small differences.^12^

**Table 2:** Efficacy outcomes

	Treatment combination			Treatment strategy			
	Sofosbuvir–velpatasvir (n=313)	Sofosbuvir–daclatasvir (n=311)		Standard of care (n=154)	Induction and maintenance (n=156)	4-week acting plus interferon (n=156)	Response-guided therapy (n=158)
**Primary outcome**							
Number assessable	307 (98–1%)	302 (97–1%)		150 (97–4%)	152 (97’4%)	152 (97–4%)	155 (98–1%)
SVR 12 weeks after end of first-line treatment	292/307 (95·1%)	294/302 (97·4%)		148/150 (98·7%)	151/152 (98.7%)	143/152 (94·1%)	144/155 (92·9%)
Primary efficacy comparison (90% CrI)	1 (ref)	2·2% (–0·2 to 4·8); P=0·069		1 (ref)	0·6% (–1·1 to 2·7); P=0·26	·4·5% (–8·3 to –1·3); P=0·99	–5·7% (–9·6 to –2·3); P=1·00
Stratification subgroup							
Genotypes 1–5	152/162 (93·8%)	153/159 (96·2%)		78/78 (100·0%)	79/80 (98·8%)	73/82 (89·0%)	75/81 (92·6%)
Genotype 6	140/145 (96·6%)	141/143 (98·6%)		70/72 (97·2%)	72/72 (100·0%)	70/70 (100·0%)	69/74 (93·2%)
**Secondary outcomes**							
SVR 12 weeks after first-line and any re-treatment	305/307 (99·3%)	302/302 (100·0%)		150/150 (100’0%)	151/152 (99·3%)	151/152 (99·3%)	155/155 (100·0%)
No initial virological response*	3/311 (1·0%)	1/310 (0·3%)		0/153	2/156 (1·3%)	0/155	2/157 (1·3%)

Data are n (%) or n/N (%), unless otherwise specified. P indicates the one-sided probability that the difference versus the reference is less than zero from the Bayesian model and is not a frequentist p value. CrI=credible interval. SVR=sustained virological response. *Not possible to calculate 90% CrI or P for the strategy comparisons due to zeros in the reference and other groups.

**Table 3:** Safety outcomes

	Treatment combination			Treatment strategy			
	Sofosbuvir–velpatasvir (n=313)	Sofosbuvir–daclatasvir (n=311)		Standard of care (n=154)	Induction and maintenance (n=156)	4-week antiviral plus interferon (n=156)	Response-guided therapy (n=158)
Serious adverse events	11 (3·5%); 13 events	6 (1·9%); 6 events		4 (2·6%); 4 events	3 (1·9%); 3 events	4 (2·6%); 5 events	6 (3·8%); 7 events
Prolonged hospitalisation	11 events	5 events		4 events	3 events	3 events	6 events
Died	0	1 event		0	0	1 event	0
Other important medical condition	2 events	0		0	0	1 event	1 event
Risk difference in serious adverse events (95% CrI)	1 (ref)	–1·6% (–4·2 to 0·8); P=0·90		1 (ref)	–0·6% (–4·1 to 2·6); P=0·66	0·0% (–3·6 to 3·5); P=0·51	1·2% (–2·7 to 5·2); P=0·26
Adverse reactions	59 (18·8%); 89 events	66 (21·2%); 88 events		5 (3·2%); 5 events	6 (3·8%); 7 events	109 (69·9%); 160 events	5 (3·2%); 5 events
Risk difference in adverse reactions (95% CrI)	1 (ref)	2–3% (–1·9 to 6·3); P=0·14		1 (ref)	0·6% (–3·4 to 4·8); P=0·38	66·8% (59–2 to 74·0); P<0·0001	–0·1% (–3·9 to 3·9); P=0·51
Changed study drugs due to adverse event	4 (0·6%); 5 events	0		0	0	4 (2·6%); 5 events	0
Grade 3 or 4 adverse events	5 (0·8%); 5 events	0		0	1 (0·6%); 1 event	3 (1·9%); 3 events	1 (0·6%); 1 event
3	5 events	0		0	1 event	3 events	1 event
4	0	0		0	0	0	0

Data are number (%) of participants who had that event, number of events, or both, unless otherwise indicated. P indicates the one-sided probability that the difference versus reference is less than zero from the Bayesian model and is not a frequentist p value. CrI=credible interval.

## Data Availability

Data access will follow the Oxford University Clinical Research Unit data sharing policy, which is based on a controlled access approach with a restriction only on data release that would compromise an ongoing trial. All individual participant data collected during the trial, after de-identification, will therefore be available (with data dictionaries), as will the trial protocol and statistical analysis plan, from immediately following publication with no end date. Anyone wishing to access the data should contact dac@oucru.org to gain access; data requestors will need to complete a short data access request form (available from https://www.oucru.org/data-sharing-policy/), which will be reviewed and approved by the OUCRU Data Access Committee, and sign a data access agreement.

## Research in context

### Evidence before this study

WHO recommends three alternative first-line treatments for treatment of chronic hepatitis C; sofosbuvir–daclatasvir, sofosbuvir–velpatasvir, and glecaprevir–pibrentasvir. Sofosbuvir–daclatasvir and sofosbuvir–velpatasvir are widely available in low-income countries through generic suppliers and countries vary in their preference for first-line therapy. However, prices vary widely and remain a barrier to access to care in many settings. We searched PubMed and Embase for articles published between Jan 1, 2010, and Dec 31, 2024, using the terms “hepatitis”, “sofosbuvir”, “daclatasvir”, “velpatasvir”, “glecaprevir/pibrentasvir”, “trials”, and “RCT”. We found that there have been no previous head-to-head studies to compare the efficacy of these first-line treatments. As national efforts make progress in scaling treatment, elimination of hepatitis C will be challenged by a small but substantial proportion of patients who require shorter or supervised therapy to be cured. There is a limited evidence base to inform treatment in these groups, a gap highlighted in previous WHO guidelines.

### Added value of this study

This study is the first head-to-head study of WHO recommended hepatitis C therapy, comparing two options widely available in low-income settings. We found that sofosbuvir–daclatasvir was non-inferior to sofosbuvir–velpatasvir. We demonstrate the high efficacy of the treatment combinations studied and, contrary to expectation, a 93% probability that sofosbuvir–daclatasvir was superior to sofosbuvir–velpatasvir in this trial. In addition to the primary comparison of sofosbuvir–velpatasvir and sofosbuvir–daclatasvir, the factorial design allowed exploration of possible strategies that might be suitable for individuals who find 12-week therapy challenging because of either lifestyle or cost. This study found high efficacy (>90%) with three novel potential treatment strategies, all of which were non-inferior to standard of care.

### Implications of all the available evidence

This study finds evidence to support the use of sofosbuvir–daclatasvir and sofosbuvir–velpatasvir in first-line treatment options. These data will be valuable to national programmes procuring treatment. For most countries scaling treatment, simplicity and standardised treatment is key. For settings with more advanced elimination programmes, the alternative strategies explored in this study can inform more individualised therapeutic decisions for those struggling to access care (eg, through supervised therapy).
