# Supplementary material for: Treatment options to support the elimination of hepatitis C: an open-label, factorial, randomised controlled non-inferiority trial
Source: Lancet. Author manuscript; Available in PMC 2026 Aug 10. (PMC7619345; doi:10.1016/S0140-6736(25)00097-2)
Supplement: Supplementary file 1 [file EMS217199-supplement-supplementary_file.pdf]

THE LANCET

Supplementary appendix

This appendix formed part of the original submission and has been peer reviewed. We post it as supplied by the authors.

Supplement to: Cooke GS, Hung LM, Flower B et al. Treatment options to support the elimination of hepatitis C: an open-label, factorial, randomised controlled non-inferiority trial. *Lancet* 2025; published online May 7. https://doi.org/10.1016/S0140-6736(25)00097-2.

Treatment options to support the elimination of hepatitis C in Viet Nam: an open label, factorial randomised controlled non-inferiority trial: Supplementary Appendix

Table of Contents **Page**

List of Investigators 2

Supplementary Methods
i. Full Inclusion Criteria 3
ii. Full Exclusion Criteria 3
iii. Determination of HCV genotype 4
iv. Management of HIV drug interactions 4
v. Statistical Monitoring 4
vi. Analysis 5

Figure S1 Trial flow 7
Figure S2 Subgroup analyses for first-line SVR12 8
Figure S3 Probability of cure by baseline HCV VL (a) in those randomised to SOF/DCV (b) in those randomised to SOF/VEL (c) difference SOF/DCV vs SOF/VEL 12
Figure S4 Undetectable HCV VL over time by (a) regimen (b) strategy 14
Figure S5 Mean HCV VL over time by (a) regimen (b) strategy 16
Figure S6 ACCEPT analysis of primary outcome (SVR12) by (a) regimen (b) strategy 18

Table S1 Full Baseline Characteristics 19
Table S2 (a) DAA exposure(b) RGT treatment duration 21
Table S3 Adherence (a) by treatment combination (b) by treatment strategy (c) by treatment strategy over time 22
Table S4 Sensitivity analyses for primary endpoint (a) by treatment combination (b) by treatment strategy 23
Table S5 SVR12 by factorial randomisation 24
Table S6 Subgroup analyses for primary endpoint (a) by treatment combination (b) by treatment strategy 25
Table S7 SVR12 by duration of treatment in RGT 29
Table S8 Association between SVR12 and genotype 30
Table S9 Details of SVR12 failures (a) overall (b) by participant 31
Table S10 Serious adverse events 33
Table S11 Grade 3/4 AEs 34
Table S12 SAEs and grade 3/4 AEs considered probably or possibly related to any study drug 34
Table S13 Adverse reactions (possibly/probably/definitely related to trial drugs) 35
Table S14 Duration and grade of adverse reactions (ARs) (possibly/probably/definitely related to trial drugs) 36
Table S15 Duration and grade of specific adverse reactions (ARs) **(**possibly/ probably/ definitely related to trial drugs) in the interferon arm 37
Table S16 Adverse reactions causing change to trial drugs 37
Table S17 Hematology test results 38
Table S18 Priors for analysis of the primary endpoint 39

Supplemental References 40

1. List of Investigators

Graham S Cooke [1,2]
Hung Le Manh, [3]
Barnaby Flower[1,2,4]
Leanne McCabe[5]
Hang Vu Thi Kim[4]
Thu Vo Thi[4]
Thuan Dang Trong[4]
Dung M.D.[3]
Phuong Le Thanh M.D.[3]
Khoa Dao Bach M.D.[3]
An Nguyen Thi Chau[4]
Thach Pham Ngoc[8]
Huong Vu Thi Thu [8]
Tuyen Nguyen Kim[7]
Azim Ansari[4,6]
Chau Le Ngoc[4]
Quang Vo Minh[3]
Phuong Nguyen Thi Ngoc[4]
Thao Le Thi[4]
Tran Nguyen Bao[4]
Evelyne Kestelyn[4,6]
Cherry Kingsley [1,2]
Rogier Van Doorn [6,7]
Motiur Rahman [4]
Sarah Pett [5]
Guy Thwaites[3,4,6]
Eleanor Barnes[6,9]
Jeremy N. Day, [3,4,10]
Chau Nguyen Van Vinh [3]
A. Sarah Walker [5,6]

Affiliations
1 Department of Infectious Disease, Imperial College London, UK
2 NIHR BRC Imperial College NHS Trust, London, UK
3 Hospital for Tropical Diseases, Ho Chi Minh City, Vietnam
4 Oxford University Clinical Research Unit (OUCRU), Ho Chi Minh City, Vietnam
5 MRC Clinical Trial Unit at UCL, University College London, UK
6 Nuffield Department of Medicine, Oxford University, Oxford, UK
7 Oxford University Clinical Research Unit (OUCRU), Hanoi, Vietnam
8 National Hospital of Tropical Diseases, Hanoi, Vietnam
9 NIHR BRC Oxford University NHS Trust, Oxford , UK
10 Department of Clinical Microbiology and Infection, Royal Devon University Healthcare, UK

2.Supplementary Methods

i. Full inclusion criteria

1. Aged ≥18 years
2. Prior evidence of Hepatitis C virus infection (evidence from clinical documentation, detected HCV antibody, HCV viraemia, qualitative viral RNA or HCV genotype.) AND at least one detectable (>LLOQ) HCV viraemia within 60 days prior to the enrolment visit (by quantitative HCV RNA, qualitative assay or HCV genotype), with no subsequent undetectable results. This latter result may come from a sample taken at the screening visit.
3. Laboratory tests at the screening visit or within 60 days of enrolment/randomisation:
   a. Creatinine clearance (estimated using Cockcroft-Gault) ≥30ml/min
   b. TSH within reference range (0.35-4.94mIU/L)
4. Mild-moderate liver disease: No evidence of significant liver fibrosis resulting from any aetiology, defined as
   EITHER
   (a) FIB-4 score of ≤1.45, equivalent to F0-F2,[1,2] within 180 days prior to planned enrolment, without transient elastography
   OR
   (b) If not meeting (a), valid Fibroscan score ≤9kPa (based on at least 10 readings, performed by an experienced (as evidenced by CV and/or training logs) technician), equivalent to F0-F2,[3] within 180 days prior to planned enrolment,
   OR
   (c) biopsy consistent with mild fibrosis (Ishak score <=2/6) within 180 days prior to planned enrolment
5. HIV-uninfected or, if HIV-infected, stable on antiretroviral therapy for >6 months (not necessarily on same regimen throughout), with HIV viral load <50 copies/ml at the screening visit, and currently taking HIV treatment compatible with all possible trial treatment options (SOF/DCV +/- PEGIFN), SOF/VEL +/- PEGIFN), with no requirement for study drug dose adjustment.
6. HBsAg negative or, if HBsAg positive, then stable on an oral nucleos(t)ide analogue (tenofovir or entecavir).
   a. Participants co-infected with HCV and HBV fulfilling the standard criteria for HBV treatment and already commenced on treatment will continue to receive nucleos(t)ide analogue treatment. Participants who are HBsAg positive but not requiring HBV treatment will commence tenofovir disoproxil fumarate prophylaxis one week prior to DAA treatment, until 12 weeks after end of treatment, in keeping with EASL[4] and local guidelines.
7. Written informed consent obtained from the participant.

ii. Full exclusion criteria

1. No previous hepatitis C treatment failure with DAA based therapy.
2. Unidentified HCV genotype after repeated sequencing attempts.
3. Any condition in the judgement of the investigator which might limit the participant's life expectancy within the duration of the study (e.g. advanced cancer).
4. Any disorder or circumstance which in the opinion of the investigator may have a significant negative impact on the ability of the participant to adhere to the trial regimen.
5. Disorder which may cause ongoing liver disease including, but not limited to, ongoing alcohol misuse.
6. Currently receiving medication known to interact with study medications, for which avoidance of co-administration or dose adjustment for SOF, DCV, VEL or PEG-IFN, would be recommended in the Summary of Product Characteristics. This includes the antiretroviral drugs efavirenz and atazanavir/ritonavir.
   a. HIV-infected individuals taking these medications may be included in the trial providing they can be switched to a suitable WHO-recommended ARV regimen. For example, efavirenz and atazanavir/ritonavir may be substituted with dolutegravir, raltegravir or lopinavir/ritonavir. Any switch should occur at least one month before randomisation, to ensure the new regimen is well-tolerated and the viral load remains suppressed. Participants requiring such a switch will continue on the new regimen for the duration of the trial. All ARV drug costs will be covered.
7. Participants currently using amiodarone or digoxin (including participants with permanent pacemakers).

8. History of severe pre-existing cardiac disease, including unstable or uncontrolled heart disease, in the previous six months
    a. Severe pre-existing cardiac disease defined as history of congestive heart failure, previous myocardial infarction, symptomatic angina in preceding 6 months, or life threatening arrhythmia (including complete heart block).
9. Abnormal ECG finding at screening in a participant with pre-existing mild-moderate cardiac disease that in the opinion of the investigator means they should not be enrolled.
10. Any pre-existing condition that may be worsened by use of PEGylated-interferon, including deranged thyroid function, autoimmune hepatitis, severe retinopathy and existence of, or history of severe psychiatric illness.
    a. Participants with thyroid disease may be eligible providing any thyroid abnormalities (TSH, T4) are adequately controlled prior to initiation of treatment.
    b. All patients will undergo fundoscopy at screening by the study doctor. Patients with diagnosed or confirmed retinopathy at screening (including macular oedema, retinal artery or vein thrombosis, retinal haemorrhage, cotton wool spots, optic neuritis, papilloedema and retinal detachment) will be excluded from the trial if it is the opinion of the investigator that there is significant risk of this deteriorating if the patient were randomised to receive PEG-IFN.
11. Use of other investigational products in clinical studies within 60 days of screening.
12. Pregnant or breastfeeding females.

iii. Determination of HCV genotype

Viral RNA was extracted from 200 µl plasma using Quick RNA Viral Kit (Zymo Research, United States). The viral RNA was reverse transcribed using the Superscript III Reverse Transcriptase protocol. The resulting cDNA was used to amplify 236 bp 5'UTR, 464 bp Core, and 377 bp NSB5 regions using primers through nested PCR. Amplicons[5] were run on 1.5% agarose gel (Merck, Germany) and purified using the Agencourt AMPure XP (Beckman Coulter, United States) according to the manufacturer's protocol. Purified amplicons were sequenced in both directions using Big Dye Terminator Cycle Sequencing Ready Reaction Kit on Applied Biosystems 3130xl Genetic Analyzer (Thermo Fisher Scientific, United States).

The nucleotide sequences from both forward and reverse directions were assembled, and a consensus sequence was generated. The consensus sequence was then blasted to the NCBI database to identify the HCV genotypes and sub-genotypes. Three regions, including 236 bp 5'UTR (position 78 to 314), 464 bp Core sequence (position 288 to 752) and 377 bp NS5B (position 8259 to 8636) of GenBank sequence AY051292 were combined to determine HCV genotypes and sub-genotypes of the sample.

iv. Management of HIV drug interactions and HBV

If an HIV-infected individual was on a contraindicated antiretroviral regimen with controlled HIV infection (undetectable HIV viral load) and it was considered safe that they should switch away from this for the duration of the DAA therapy, they were switched at least 1 month before planned enrolment in the study to ensure the new HIV regimen was well tolerated and the HIV viral load remained fully suppressed. If HIV-infected individuals were not already taking it, the fixed-dose combination of tenofovir disoproxil fumarate, lamivudine and dolutegravir (TLD) was provided within the trial to all HIV co-infected participants on efavirenz. If an individual was not able to tolerate dolutegravir, they were not eligible for randomisation (unless they were taking other compatible drugs). Individuals already on second-line antiretroviral therapy were eligible only if their regimen was already compatible with the DAAs used in the trial.

HBsAg positive individuals who were not stable on a nucleos(t)ide analogue therapy were excluded from this trial (see above). Participants coinfected with HCV and HBV, fulfilling the standard criteria for HBV treatment and already commenced on treatment, continued to receive nucleos(t)ide analogue treatment (tenofovir or entectavir). Participants who were HbsAg positive but not requiring HBV treatment commenced tenofovir prophylaxis one week prior to DAA treatment, until 12 weeks after end of treatment, following European and local guidelines. Tenofovir was provided for the duration of the trial for all patients who were HbsAg positive.

v. Statistical Monitoring

Statistical monitoring of each randomised arm, stratified by genotype, followed a Bayesian approach to detect and stop recruitment into unsuccessful treatment strategies, defined by having >0.95 posterior probability that the true SVR12 rate was less than 90% (non-comparative, within randomised arm).[6] In addition the Data

Monitoring Committee also monitored comparisons between randomised drug and strategy arms as standard. They held three meetings to review data; no arms were stopped.

vi. Analysis

All analyses were predefined in a Statistical Analysis Plan. Baseline values for all measurements were those recorded at screening or enrolment on the screening, baseline and laboratory tests forms, taking the closest to enrolment and using values after enrolment only if they were from the day of enrolment and before the participant took their first dose of study drug. SVR was measured from the scheduled end date of the most recent treatment.

Failure was defined as described in the main Methods; in addition

- Any participant who failed on first-line treatment and chose not to be retreated was counted as a failure for retreatment.
- Any participant lost to follow up after single HCV RNA>2000 IU/ml was counted as a failure, although every attempt was made to obtain local RNA results for any such participant. Any participant lost to follow up after single RNA>LLOQ but <2000 IU/ml was not counted as a failure.
- Any death with detectable RNA was counted as a failure.
- Participants whose confirmatory test at EOT+12 was >LLOQ were counted as failures. However, where a single HCV RNA at EOT+12 was >LLOQ but prior and subsequent values are <LLOQ, it is implausible that genuine failure has occurred. Therefore any participant with a single HCV VL>LLOQ at EOT+12 weeks, but one or more prior values post EOT <LLOQ and all subsequent values <LLOQ (including those performed by the site as part of standard clinical practice post EOT+12) was considered to have achieved SVR12.

The date of treatment failure was the date of the first failure result (which was then subsequently confirmed). In terms of the Estimands framework[7] (applied retrospectively), intercurrent events were treated as follows

- Death: hypothetical strategy (because death expected to be extremely rare and unrelated to Hepatitis C infection in the population with mild-moderate disease recruited into the trial)
- All treatment-related intercurrent events: treatment policy strategy (analogous to intention-to-treat)

Methods for handling missing data in estimation were:

- Patients excluded due to being lost to follow-up before EOT+12 weeks with last HCV VL undetectable were missing completely at random.
- Patients defined as "failures" due to being lost to follow-up before EOT+12 weeks after observing a single HCV VL >2000 IU/ml (as defined above) would have met the outcome definition of failure had they not been lost.

The Statistical Analysis Plan specified that if missing SVR12 data were <10%, multiple imputation would not be performed, but as well as the primary analysis restricted to those with assessable SVR12, and analyses treating all those lost to follow-up as either successes or failures would be performed. However, as missing SVR12 was very low (15/624, 2.4%) and evenly distributed across arms (**Figure 2**), this was not done.

The priors for the final efficacy analysis are shown in **Table S18** (details in[6]). Efficacy analysis was conducted as follows. The difference in percentages between randomised arms was compared by first performing logistic regression using Bayesian methods and then estimating the risk difference by the average difference in risk between the arms being analysed. This was done by calculating Y[i,1] and Y[i,0] for each individual i and therefore the average of Y[i,1] – Y[i,0], via Markov chain Monte Carlo methods. The primary priors were the uninformative priors in **Table S18**. Secondary analyses used the sceptical and enthusiastic priors. Secondary analyses included Bayesian analysis with 95% credible intervals with uninformative priors only, and frequentist analysis with 95% confidence intervals (as above by calculating a risk difference and 95% confidence interval obtained from marginal effects after a logistic regression). These Bayesian analyses were also presented using ACCEPT (Acceptability Curve Estimation using Probability above Threshold) methods (**Figure S6**).

Subgroup effects were assessed for the primary outcome using heterogeneity tests in frequentist logistic regression (penalised logistic regression (Stata firthlogit) where cure rates were 100% in one or more subgroups and this would converge (penalisation one-half of the logarithm of the determinant of the information matrix)). In addition to the subgroup analysis for the factorial randomisations implicit within the trial design, the protocol specified 10 subgroup analyses by baseline HCV VL (treated as a continuous interaction and also considering a dichotomisation at 6,000,000 IU/ml), genotype 6 vs non-6 (also splitting non-6 into 1, 2 other if numbers

allowed), presence of viral quasispecies including significant baseline resistance-associated substitutions (RASs) (not performed as assays ongoing), age (related to immune health), sex, HIV co-infection, IL28 polymorphisms (CC v non-CC), BMI, early HCV RNA treatment responses (not specified in the protocol but considered at day 7 and 14 depending on randomised arm given different assessment schedules (**Figure S1**)), and previous (failed) treatment with interferon-alpha with/out ribavirin (not performed as no participants had received previous treatment, **Table S1**) (nine subgroup analyses performed). The Statistical Analysis Plan added an additional subgroup analysis by centre (total 10 pre-specified subgroup analyses). Two additional subgroups were considered exploratory by the investigators (previous illicit substance use and Fibroscan) and three were requested by a reviewer (calendar time; pre-COVID lockdown vs two periods subsequently), missing first-line visits and missing any DAA doses. Continuous variables were considered as continuous interactions using fractional polynomials and also splitting into approximate terciles unless otherwise specified. [8]

The primary analysis was modified intention-to-treat including only participants in whom the primary endpoint could be evaluated. 15 (2.4%) participants were excluded from the primary analysis as they were lost to follow-up during first-line (one additional participant lost to follow-up during retreatment). Three were lost immediately after randomisation and never started treatment. Eight were lost during first-line treatment (at 2 weeks, 3 weeks, 4 weeks x2 (one PEG-IFN so also EOT), 7 weeks x3, 12 weeks (EOT)), and four were lost after EOT (two 4 weeks post EOT, two 8 weeks post EOT). Reasons were death (unknown cause, see main text), withdrawal of consent (n=5; no reasons provided), and could no longer be contacted (n=9; one emigrated, 8 could not be contacted).

Figure S1 Trial flow

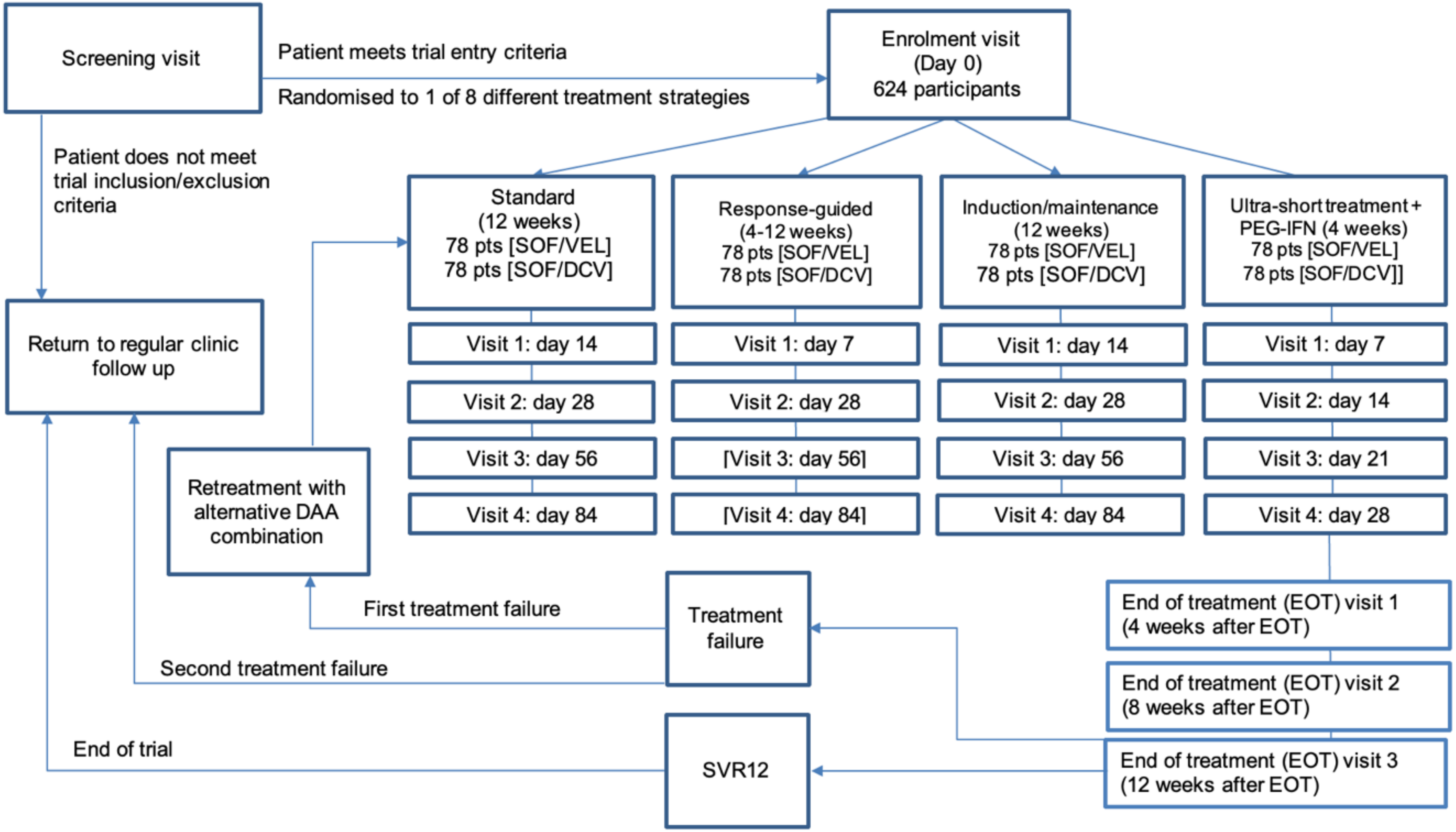

Figure S2 Subgroup analyses for first-line SVR12

a) Treatment combination comparison

Subgroup	Risk difference	95% CI		Heterogeneity p-value
Overall	2.2	-0.7	5.2	.
Strategy: IFN	3.9	-3.5	11.4	.
Strategy: RGT	1.1	-7.0	9.1	.
Baseline VL >6m IU/ml	5.5	-0.0	10.9	0.194
Baseline VL <6m IU/ml	0.9	-2.6	4.4	.
HCV genotype: non-G6	2.4	-2.3	7.1	0.701
HCV genotype: G6	2.0	-1.5	5.5	.
Age: <40 years	1.2	-3.9	6.4	0.406
Age: 40-48 years	5.1	0.2	10.0	.
Age: >48 years	0.2	-4.9	5.3	.
Sex: male	2.3	-1.1	5.7	0.909
Sex: female	2.0	-4.1	8.1	.
BMI: <21 kg/m^2	4.1	-1.1	9.2	0.677
BMI: 21-23.6 kg/m^2	1.6	-3.7	6.9	.
BMI: >23.6 kg/m^2	1.0	-4.1	6.0	.
HIV negative	2.3	-0.9	5.5	0.992
HIV positive	2.1	-5.9	10.2	.
D14 HCV VL detectable	1.2	-3.6	6.0	0.336
D14 HCV VL undetectable	3.3	-0.3	7.0	.
Hanoi	2.8	-1.5	7.1	0.667
HCMC	1.8	-2.4	5.9	.
IFNL4: CC	1.8	-1.2	4.8	0.799
IFNL4: CT/TT	5.2	-5.3	15.8	.
Illicit substance abuse	3.2	-2.4	8.8	0.787
No illicit substance abuse	1.8	-1.7	5.3	.
Fibroscan: <5kPa	5.6	-2.3	13.4	0.400
Fibroscan: 5-7kPa	2.6	-1.1	6.3	.
Fibroscan: >=7kPa	-0.6	-6.4	5.3	.
No missed visits	1.5	-1.3	4.4	0.862
Missed visits	3.0	-22.1	28.0	.
Enrolled pre-COVID-19 lockdowns	-0.2	-6.1	5.7	0.155
Enrolled between lockdowns - 01/06/2022	-1.2	-6.1	3.8	.
Enrolled after 01/06/2022	5.7	1.1	10.3	.

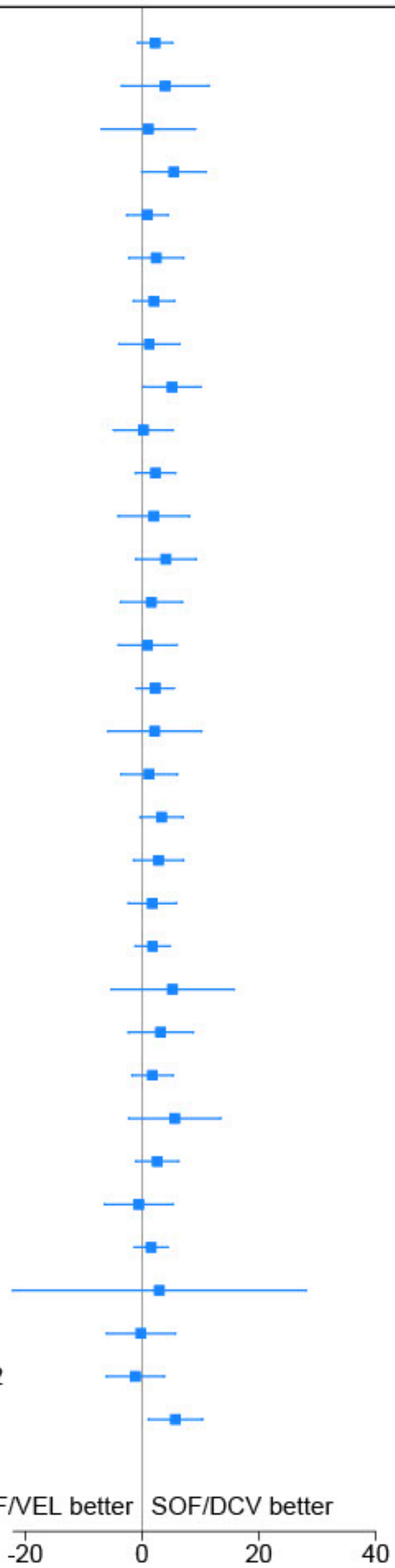

b) Treatment strategy comparison: RGT vs SOC

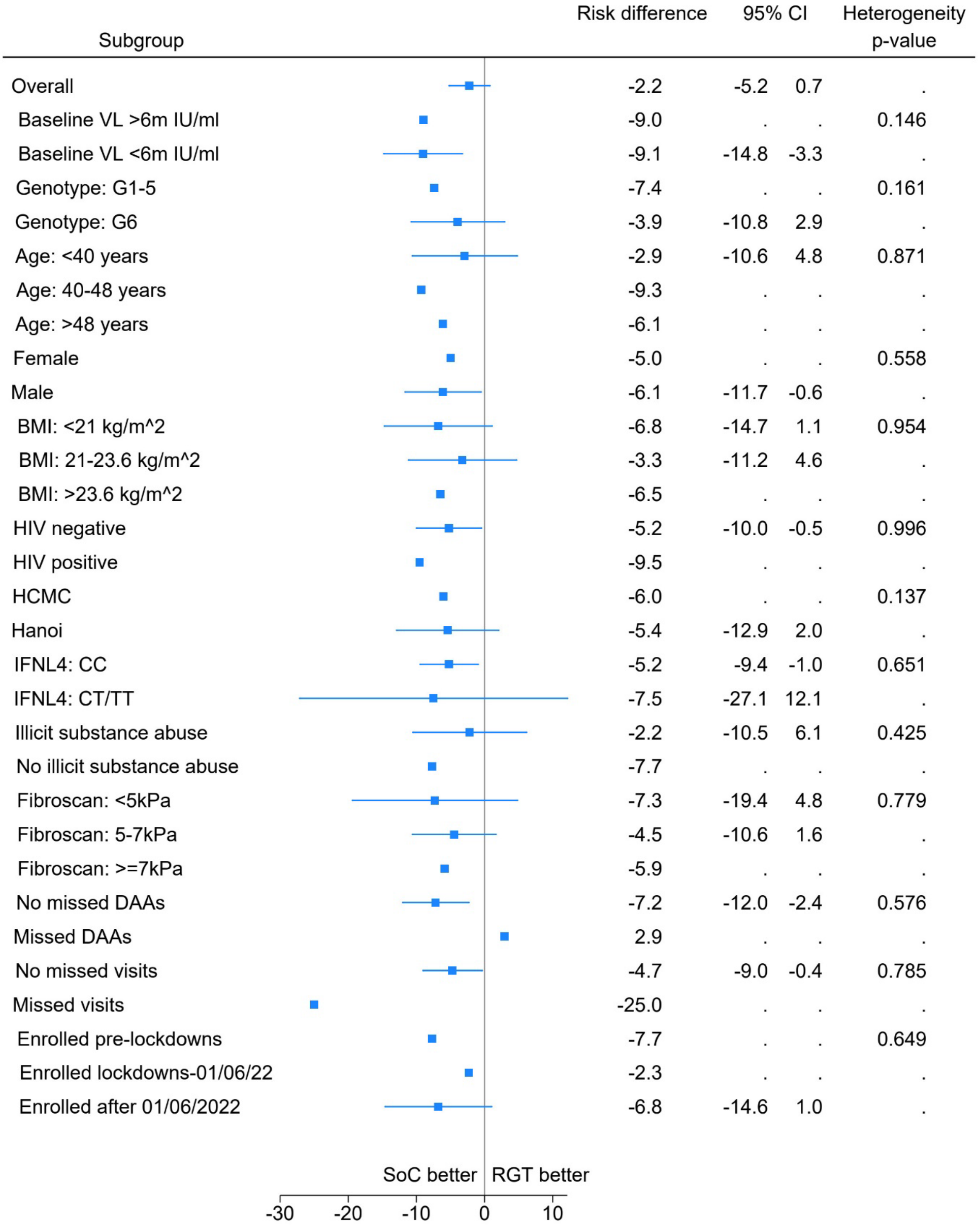

(c) Treatment strategy comparison: I/M vs SOC

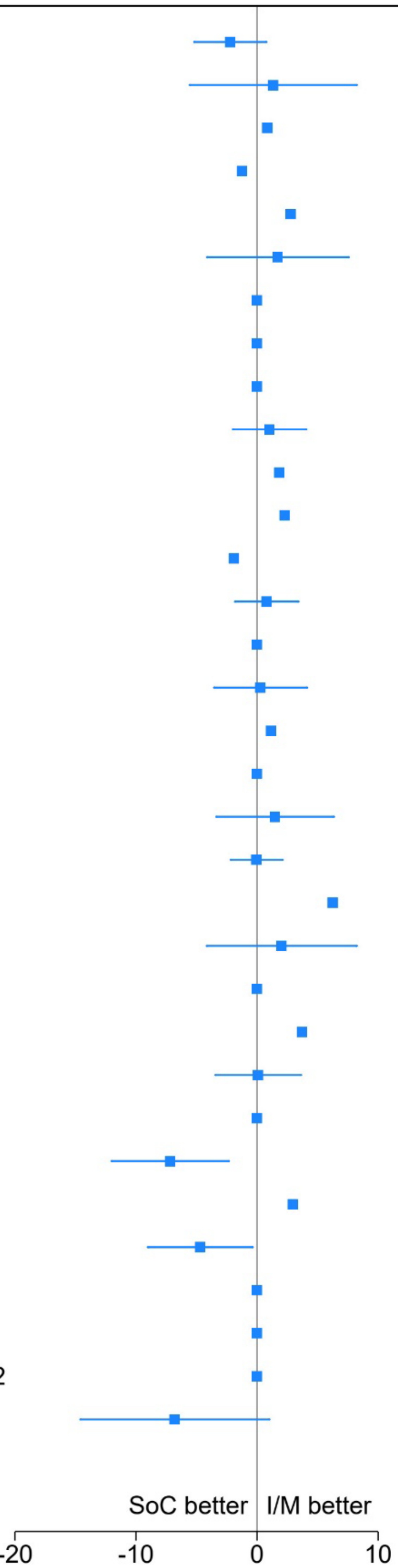

Subgroup	Risk difference	95% CI		Heterogeneity p-value
Overall	-2.2	-5.2	0.7	.
Baseline VL >6m IU/ml	1.3	-5.6	8.2	.
Baseline VL <6m IU/ml	0.9	.	.	.
Genotype: G1-5	-1.2	.	.	.
Genotype: G6	2.8	.	.	.
Age: <40 years	1.7	-4.1	7.5	.
Age: 40-48 years	0.0	.	.	.
Age: >48 years	0.0	.	.	.
Female	0.0	.	.	.
Male	1.0	-2.0	4.0	.
BMI: <21 kg/m^2	1.8	.	.	.
BMI: 21-23.6 kg/m^2	2.3	.	.	.
BMI: >23.6 kg/m^2	-1.9	.	.	.
HIV negative	0.8	-1.8	3.4	.
HIV positive	0.0	.	.	.
D14 HCV VL detectable	0.3	-3.6	4.1	0.898
D14 HCV VL undetectable	1.1	.	.	.
HCMC	0.0	.	.	.
Hanoi	1.5	-3.4	6.3	.
IFNL4: CC	-0.1	-2.2	2.1	.
IFNL4: CT/TT	6.2	.	.	.
Illicit substance abuse	2.0	-4.2	8.2	.
No illicit substance abuse	0.0	.	.	.
Fibroscan: <5kPa	3.7	.	.	.
Fibroscan: 5-7kPa	0.1	-3.4	3.6	.
Fibroscan: >=7kPa	0.0	.	.	.
No missed DAAs	-7.2	-12.0	-2.4	.
Missed DAAs	2.9	.	.	.
No missed visits	-4.7	-9.0	-0.4	.
Missed visits	0.0	.	.	.
Enrolled pre-lockdowns	0.0	.	.	.
Enrolled lockdowns-01/06/22	0.0	.	.	.
Enrolled after 01/06/2022	-6.8	-14.6	1.0	.

(d) Treatment strategy comparison: 4w-DAA/IFN vs SOC

Subgroup	Risk difference	95% CI		Heterogeneity p-value
Overall	-2.2	-5.2	0.7	.
Baseline VL >6m IU/ml	-7.9	-18.6	2.9	.
Baseline VL <6m IU/ml	-2.9	-6.8	1.1	.
Genotype: G1-5	-11.0	.	.	.
Genotype: G6	2.8	.	.	.
Age: <40 years	-1.0	-8.7	6.7	.
Age: 40-48 years	-7.4	.	.	.
Age: >48 years	-5.7	.	.	.
Female	-10.8	.	.	.
Male	-2.4	-6.8	2.0	.
BMI: <21 kg/m^2	-3.2	-10.6	4.3	.
BMI: 21-23.6 kg/m^2	-4.2	-12.0	3.7	.
BMI: >23.6 kg/m^2	-5.9	.	.	.
HIV negative	-4.3	-8.7	0.2	.
HIV positive	-5.9	.	.	.
D14 HCV VL detectable	-8.0	-17.1	1.2	.
D14 HCV VL undetectable	-3.3	-7.7	1.2	.
HCMC	-9.6	.	.	.
Hanoi	1.5	-3.4	6.3	.
IFNL4: CC	-4.7	-8.9	-0.5	.
IFNL4: CT/TT	-1.2	-18.1	15.6	.
Illicit substance abuse	-2.7	-11.5	6.0	.
No illicit substance abuse	-5.7	.	.	.
Fibroscan: <5kPa	-1.8	-11.8	8.2	.
Fibroscan: 5-7kPa	-1.6	-6.4	3.1	.
Fibroscan: >=7kPa	-11.9	.	.	.
No missed DAAs	0.1	-2.1	2.4	.
Missed DAAs	-7.6	-27.7	12.4	.
No missed visits	0.7	-1.7	3.1	.
Missed visits	-42.9	.	.	.
Enrolled pre-lockdowns	-7.9	.	.	.
Enrolled lockdowns-01/06/22	-8.5	.	.	.
Enrolled after 01/06/2022	1.4	-3.4	6.2	.

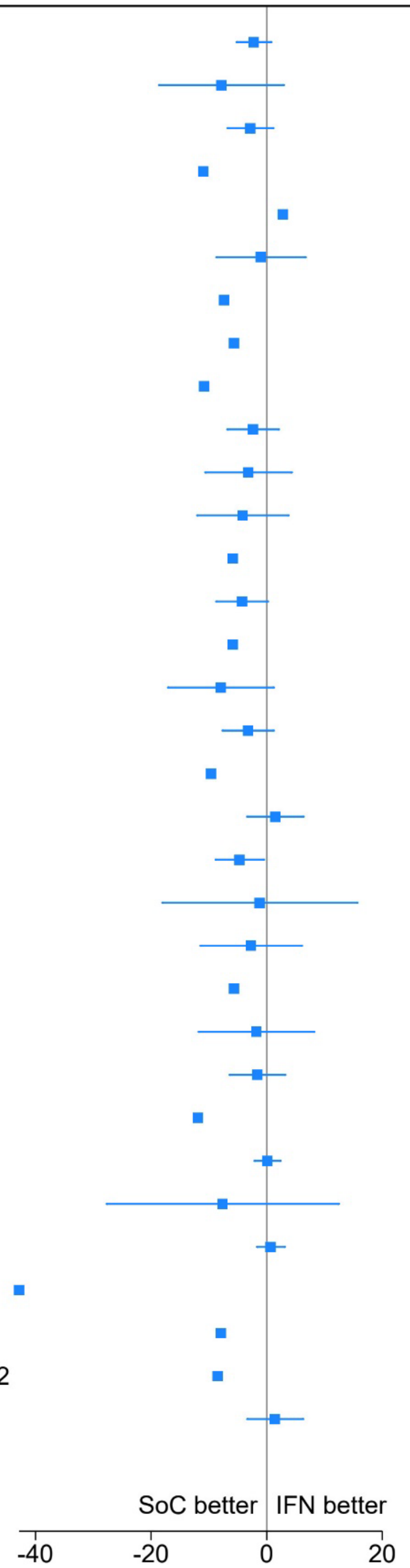

Note: showing estimable risk differences and 95% CI (some comparisons not possible due to 100% cure in some subgroups); full details of primary outcome rates also presented in **Table S6**. Subgroup analyses were prespecified in the protocol with the exception of illicit substance use and Fibroscan result (added by investigators); and calendar time (divided into periods before the first regional lockdown in each site (HCMC 21 May 2021, Hanoi 11 June 2021), up to 1 June 2022 and subsequently), missing first-line visits and missing any DAA doses, added at the request of a reviewer, see **Supplementary Methods**.

Figure S3 Probability of cure by baseline HCV VL (a) in those randomised to SOF/DCV (b) in those randomised to SOF/VEL (c) difference SOF/DCV vs SOF/VEL

(a)

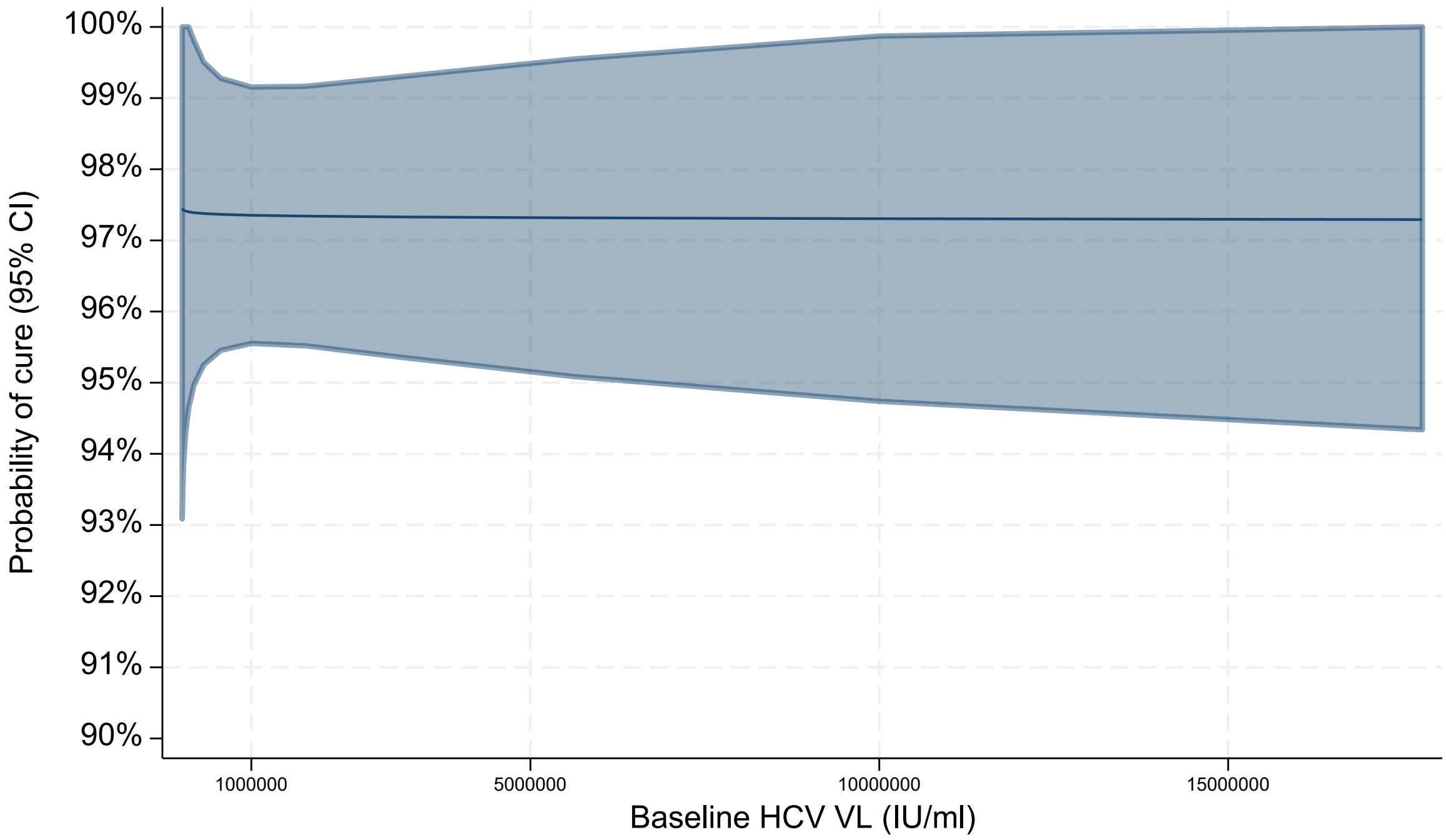

(b)

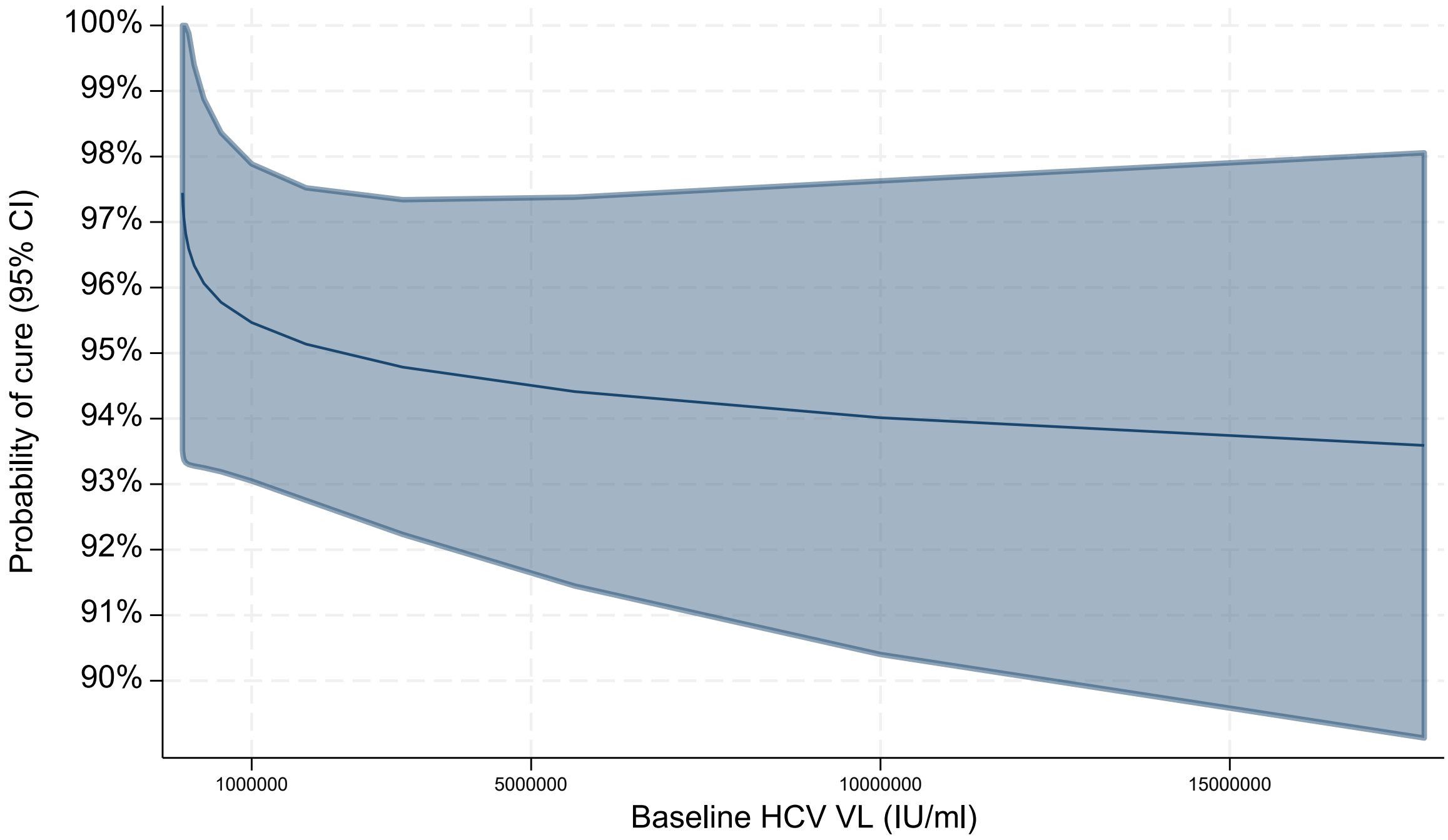

(c)

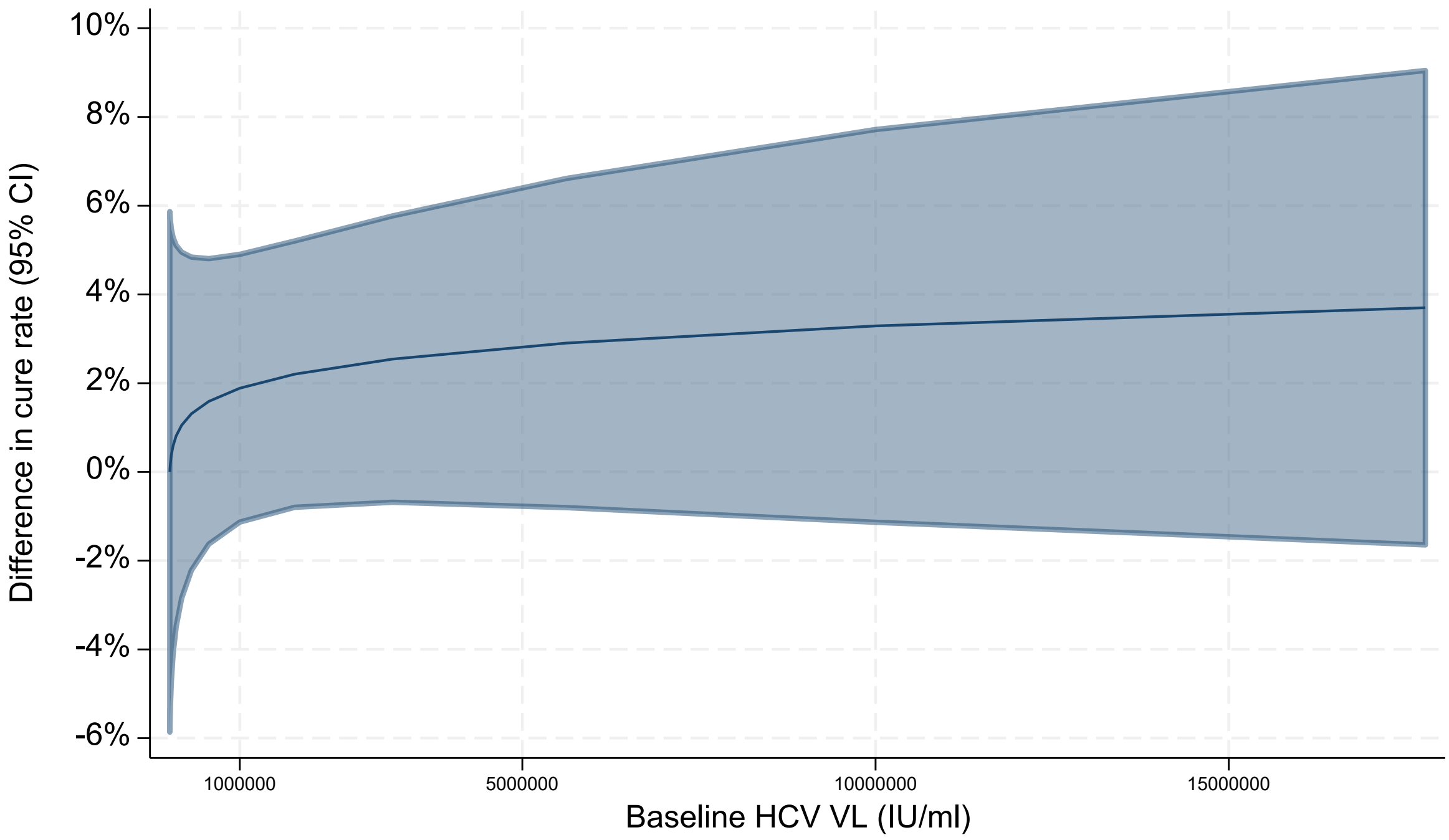

10%
8%
6%
4%
2%
0%
-2%
-4%
-6%
Difference in cure rate (95% CI)
1000000
5000000
10000000
15000000
Baseline HCV VL (IU/ml)

Figure S4 Undetectable HCV VL over time by (a) regimen (b) strategy

a)

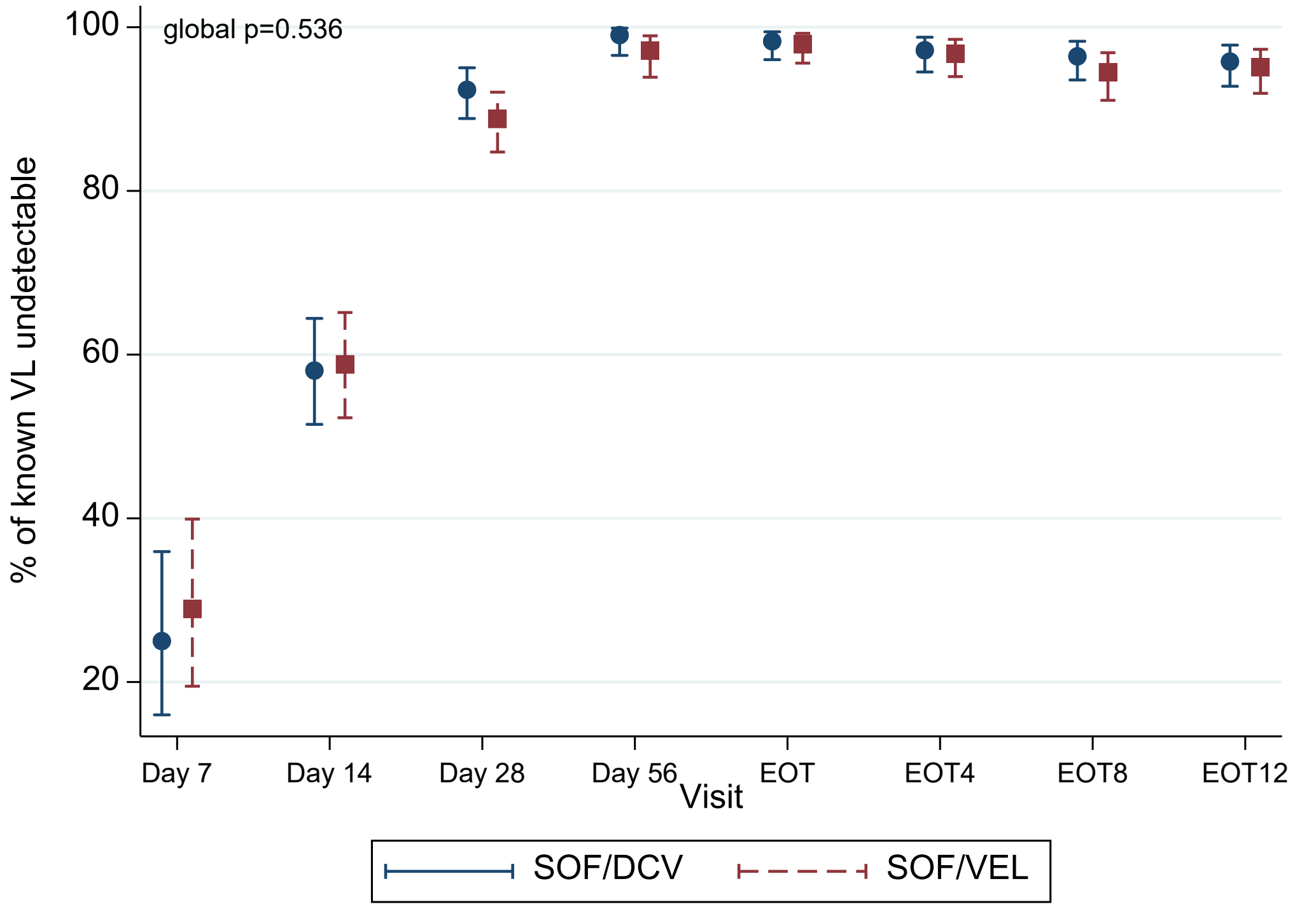

	SOF/DCV (% undetectable)	SOF/VEL (% undetectable)	Total (% undetectable)	p-value
Day 7	N=80	N=83	N=163	
	20 (25%)	24 (29%)	44 (27%)	p=0.573
Day 14	N=236	N=238	N=474	
	137 (58%)	140 (59%)	277 (58%)	p=0.864
Day 28	N=314	N=312	N=626	
	290 (92%)	277 (89%)	567 (91%)	p=0.126
Day 56	N=207	N=209	N=416	
	205 (99%)	203 (97%)	408 (98%)	p=0.157
EOT	N=291	N=294	N=585	
	286 (98%)	288 (98%)	574 (98%)	p=0.774
EOT+4	N=284	N=279	N=563	
	276 (97%)	270 (97%)	546 (97%)	p=0.777
EOT+8	N=281	N=272	N=553	
	271 (96%)	257 (94%)	528 (95%)	p=0.268
EOT+12	N=286	N=286	N=572	
	274 (96%)	272 (95%)	546 (95%)	p=0.688

Note: For participants randomised to PEG-IFN or RGT and allocated 4 weeks, their EOT visit was also their D28 visit. For participants randomised to RGT and allocated 8 weeks, their EOT visit was also their D56 visit. In all cases, D28/D56/EOT results are included twice here and in the binomial GEE.

Individual p-values calculated using chi-squared test; global p-value calculated from binomial GEE for all visits. Participants with a treatment failure are considered as having a detectable result for all possible later visits so the denominators are those with a known VL result or a previous treatment failure. Only RGT measured at Day 7 and only SOC, 4w-DAA/IFN and I/M at Day 14 (see **Figure S1**).

(b)

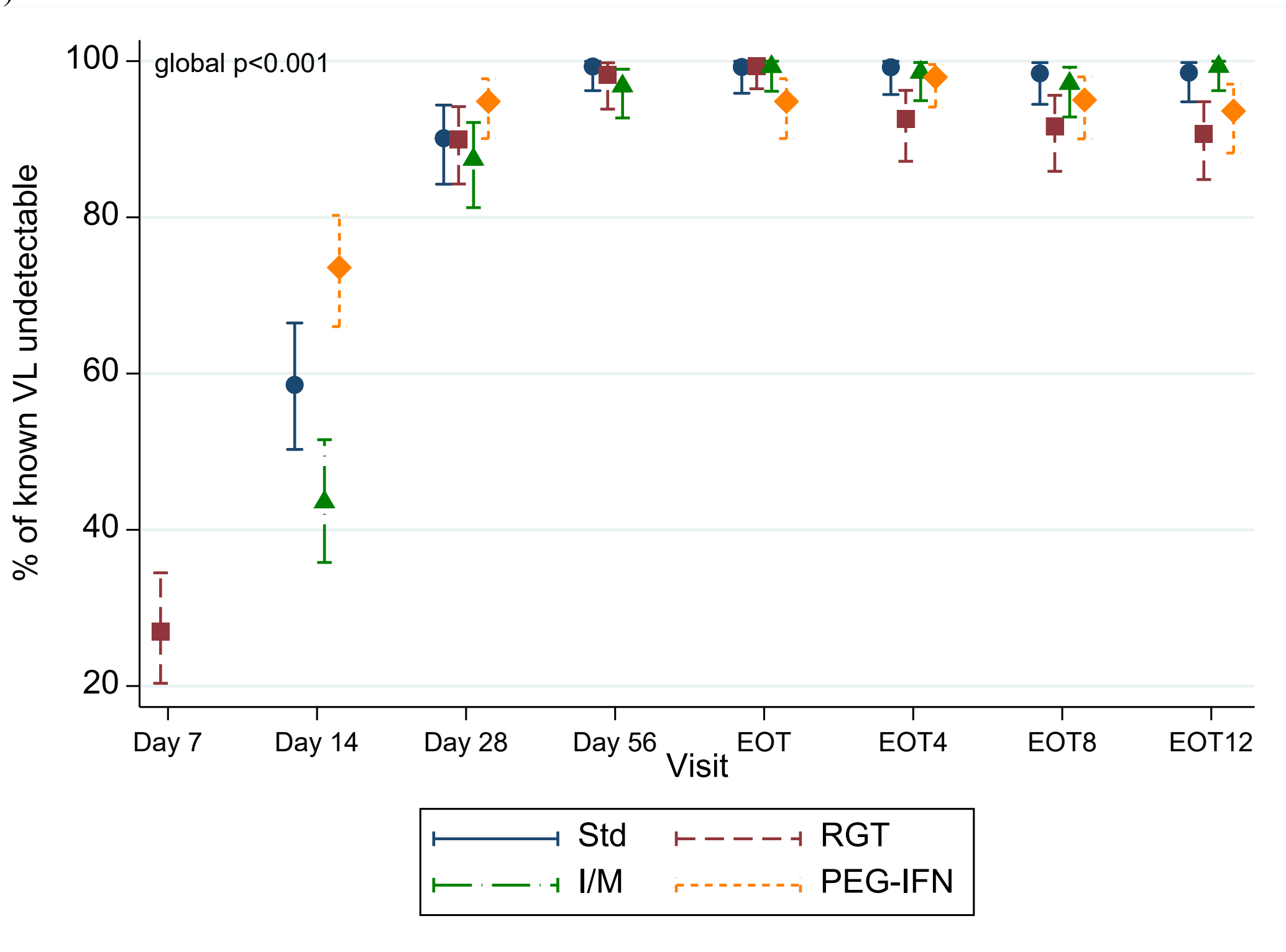

	SOC (% undetectable)	RGT (% undetectable)	I/M (% undetectable)	4w-DAA/IFN (% undetectable)	Total (% undetectable)	p-value
Day 7	-	N=163	-	-	N=163	
	-	44 (27%)	-	-	44 (27%)	-
Day 14	N=152	-	N=163	N=159	N=474	
	89 (59%)	-	71 (44%)	117 (74%)	277 (58%)	p<0.001
D28	N=152	N=160	N=159	N=155	N=626	
	137 (90%)	144 (90%)	139 (87%)	147 (95%)	567 (91%)	p=0.154
Day 56	N=145	N=114	N=157	-	N=416	
	144 (99%)	112 (98%)	152 (97%)	-	408 (98%)	p=0.357
EOT	N=133	N=155	N=142	N=155	N=585	
	132 (99%)	154 (99%)	141 (99%)	147 (95%)	574 (98%)	p=0.011
EOT+4	N=128	N=149	N=140	N=146	N=563	
	127 (99%)	138 (93%)	138 (99%)	143 (98%)	546 (97%)	p=0.008
EOT+8	N=128	N=144	N=140	N=141	N=553	
	126 (98%)	132 (92%)	136 (97%)	134 (95%)	528 (95%)	p=0.047
EOT+12	N=136	N=150	N=145	N=141	N=572	
	134 (99%)	136 (91%)	144 (99%)	132 (94%)	546 (95%)	p<0.001

Note: For participants randomised to PEG-IFN or RGT and allocated 4 weeks, their EOT visit was also their D28 visit. For participants randomised to RGT and allocated 8 weeks, their EOT visit was also their D56 visit. In all cases, D28/D56/EOT results are included twice here and in the binomial GEE.
p-values calculated using chi-squared test or Fisher's exact test for small values; global p-value calculated from binomial GEE for all visits.
Participants with a treatment failure are considered as having a detectable result for all possible later visits so the denominators are those with a known VL result or a previous treatment failure.
Only RGT measured at Day 7 and only SOC, 4w-DAA/IFN and I/M at Day 14 (see **Figure S1**).

Figure S5 Mean HCV VL over time by (a) regimen (b) strategy

a)

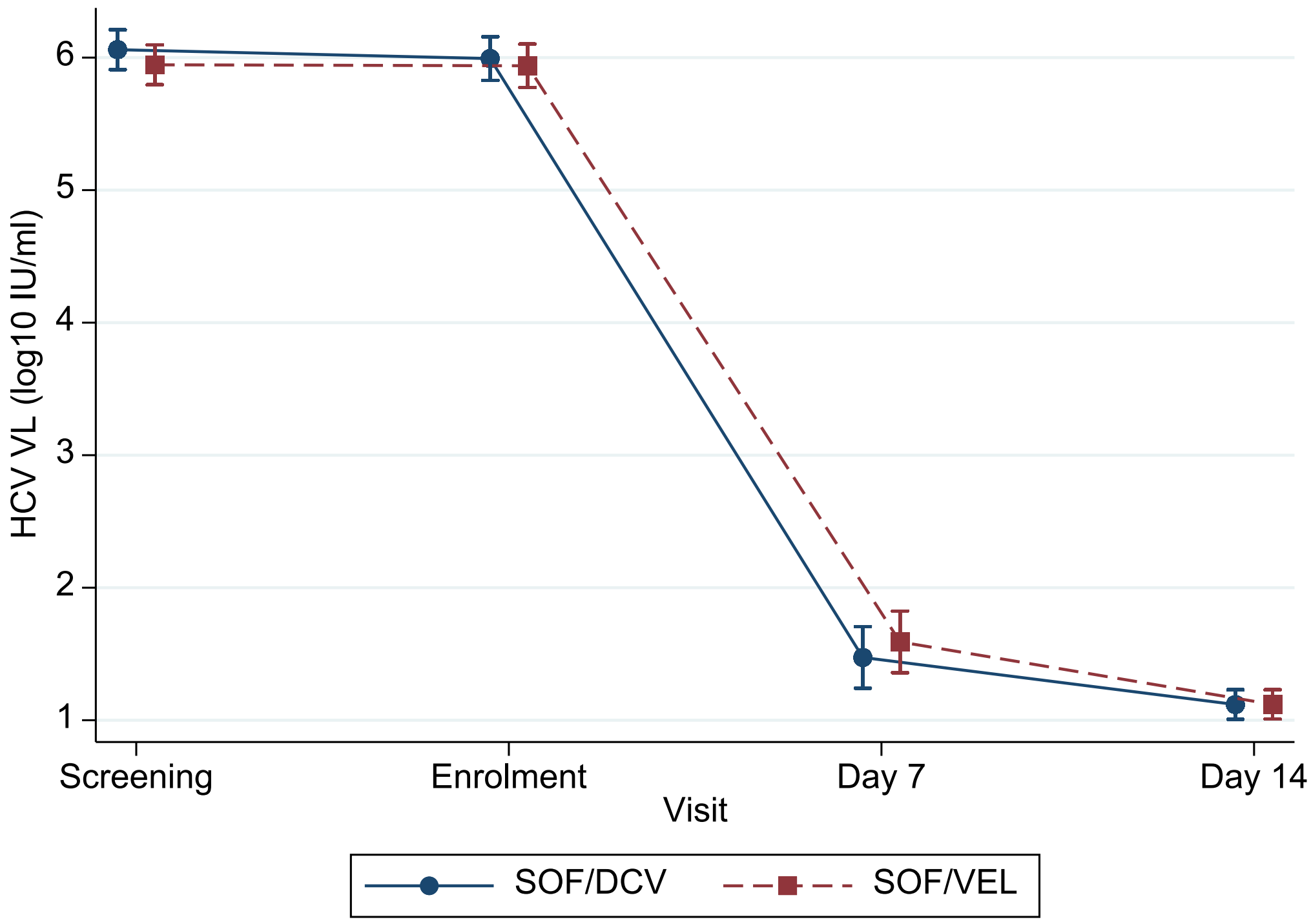

	SOF/DCV	SOF/VEL	Difference between arms	p-value
	N=76	N=79		
Day 7: mean (95% CI)	1.54 (1.34, 1.74)	1.68 (1.48, 1.87)	0.14 (-0.07, 0.35)	p=0.194
	N=231	N=229		
Day 14: mean (95% CI)	1.13 (1.03, 1.24)	1.14 (1.04, 1.24)	0.01 (-0.10, 0.11)	p=0.927

Note: Estimated using interval regression to incorporate values below limit of quantification. Only RGT measured at Day 7 and only SOC, 4w-DAA/IFN and I/M at Day 14 (see **Figure S1**).

b)

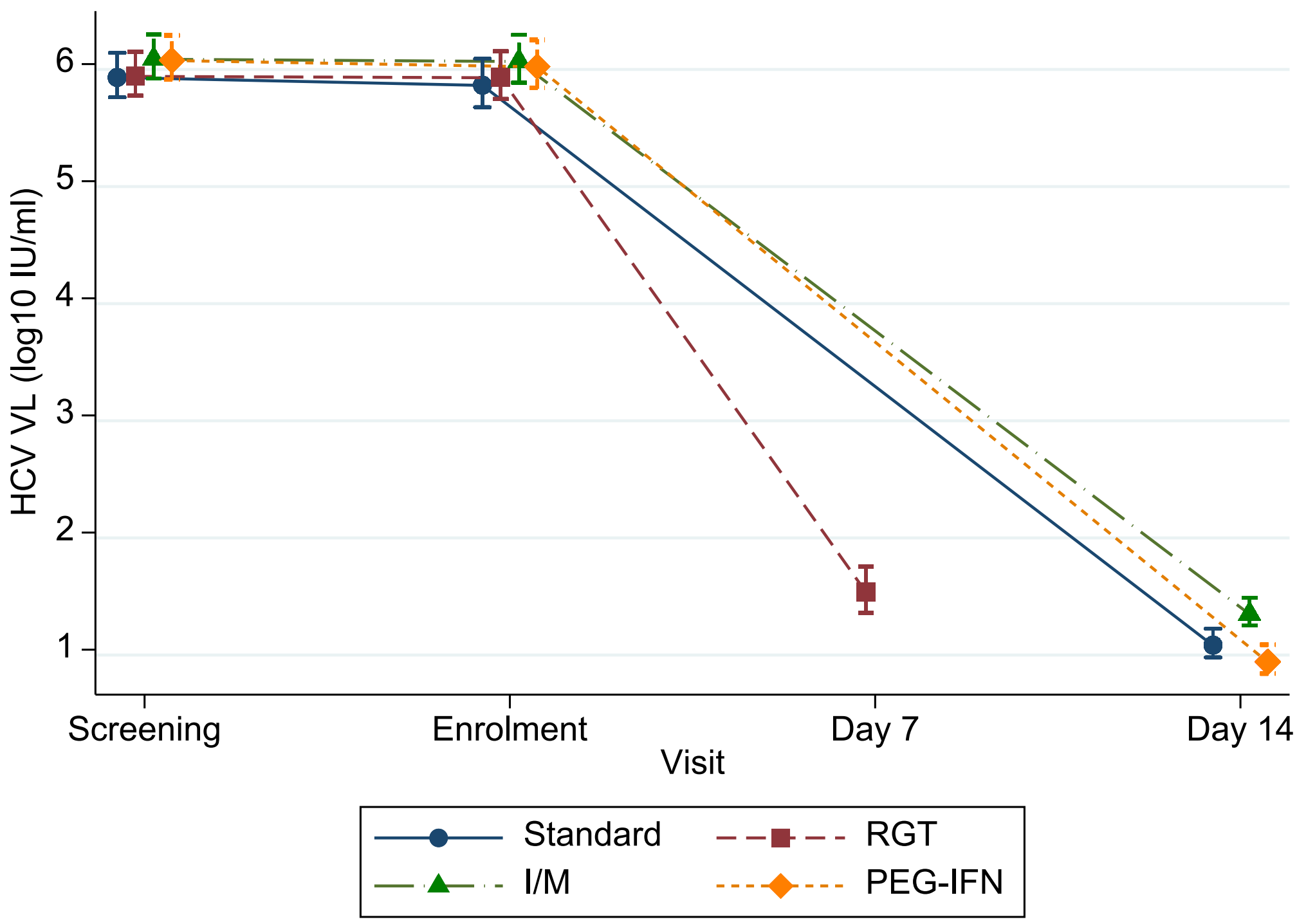

	SOC	RGT	I/M	4w-DAA/IFN
	-	N=155	-	-
Day 7: mean (95% CI)	-	1.61 (1.44, 1.78)	-	-
	N=152	-	N=156	N=152
Day 14: mean (95% CI)	1.12 (1.01, 1.24)	-	1.33 (1.22, 1.44)	0.94 (0.82, 1.05)
Difference from standard treatment (95% CI)	Ref	-	0.21<br>(0.08, 0.33) p=0.001	-0.19<br>(-0.32, -0.06) p=0.005

Note: Estimated using interval regression to incorporate values below limit of quantification. HCV VL was only measured in the RGT group on Day 7 and was not measured in the RGT group on Day 14.

Figure S6 ACCEPT analysis of primary outcome (SVR12) by (a) regimen (b) strategy
(a)

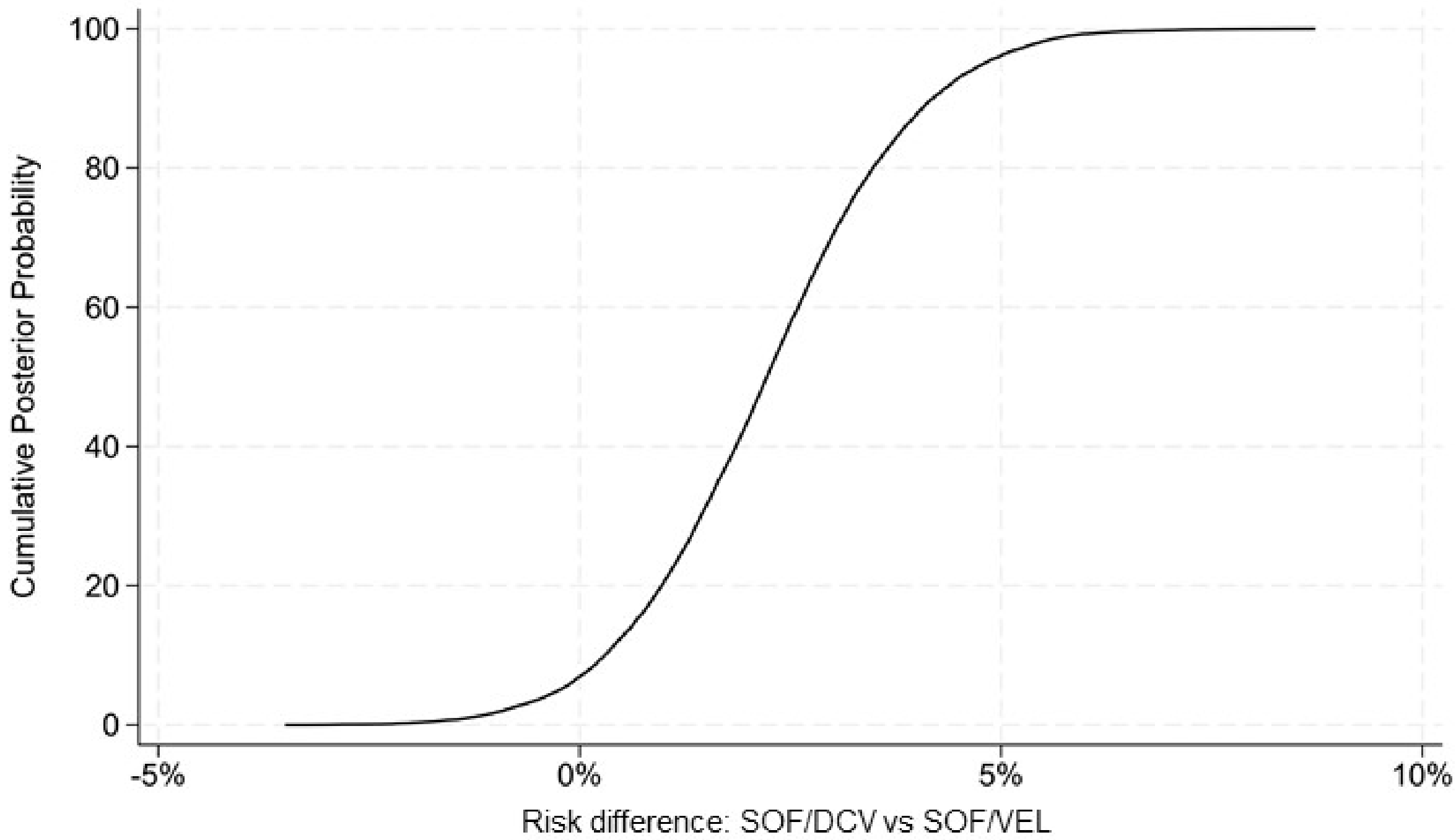

(b)

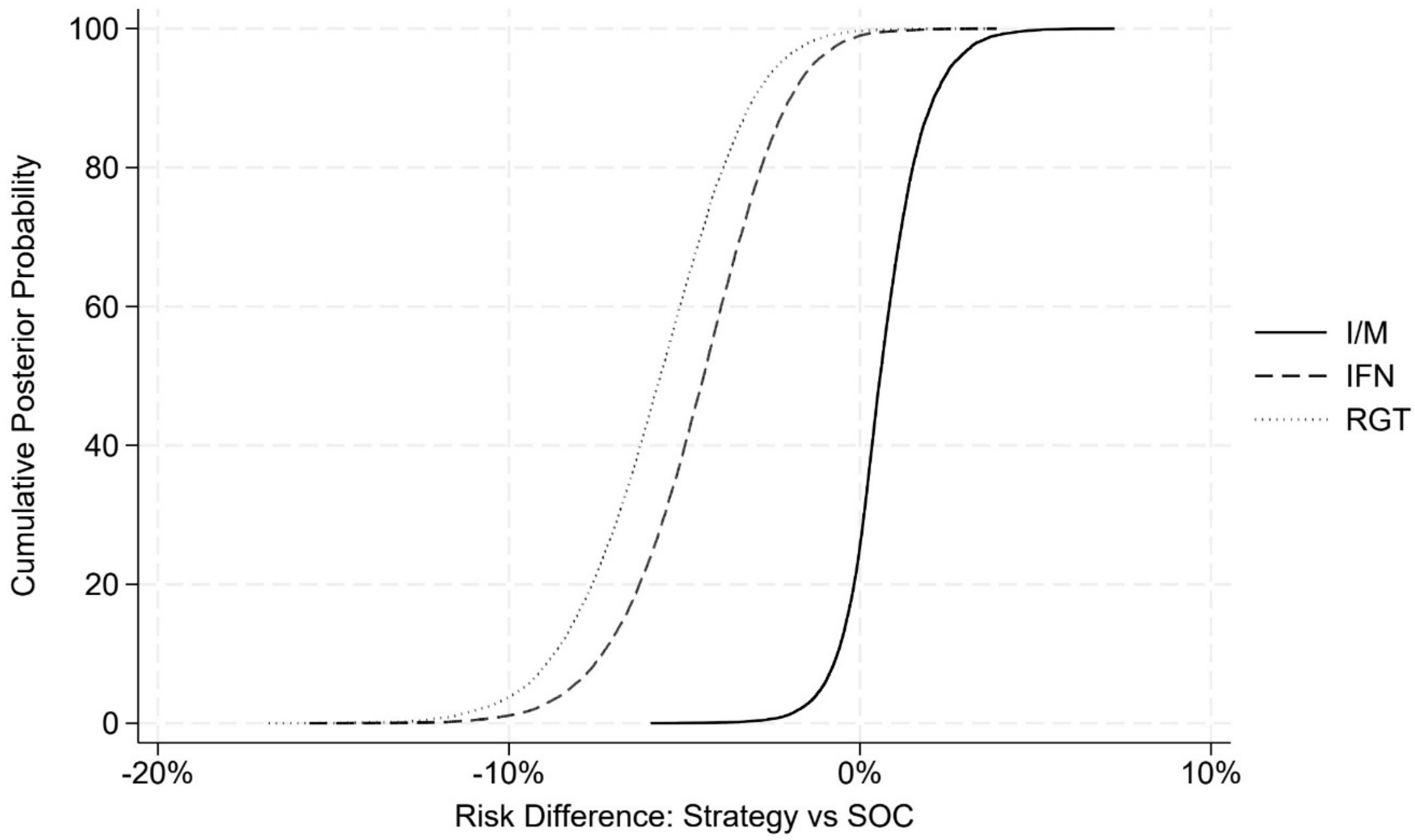

Note: ACCEPT=Acceptability Curve Estimation using Probability above Threshold[8] panels show the cumulative probability that the intervention is worse than (leads to lower cure rates) than the comparator by each risk difference. For example, there are very small probabilities that SOF/DCV is more than 2% worse than SOF/VEL, and SOF/DCV is almost certainly no more than 6% better than SOF/VEL. Risk difference corresponding to 50% probability is the central estimate of the credible interval and 90% and 95% limits correspond to $5^{th}/95^{th}$ and $2.5^{th}/97.5^{th}$ percentiles respectively.

Table S1 Full Baseline Characteristics

	Total
	N=624
Hanoi	290 (46%)
HCMC	334 (54%)
Age (years)	42 (37, 51) [18, 80]
Female	154 (25%)
Weight (kg)	59.1 (53.0, 65.8) [34.6, 90.5]
Height (cm)	164 (158, 168) [141, 185]
BMI (kg/m^2)	22.1 (20.3, 24.4) [15.2, 33.5]
HCV viral load (IU/ml)	2221075 (395702, 6962394) [18, 100000000]
HCV viral load ($\log_{10}$ IU/ml)	6.1 (1.1) [1.2, 8.0]
HCV genotype/ subgenotype	
1	257 (41%)
a	151
b	105
undetermined	1
2	49 (8%)
a	33
b	1
j	1
m	7
undetermined	7
3	22 (4%)
a	6
b	16
6	296 (47%)
a	179
e	62
h	19
i	1
l	7
p	2
undetermined	17
Fibroscan result (kPa) n=615	6.1 (5.0, 7.3) [2.9, 9.0]
FIB4 score n=9	0.9 (0.8, 0.9) [0.5, 1.3]
HIV positive	83 (13%)
HBsAg positive n=623	39 (6%)
Previous HCV treatment	
No previous HCV treatment	624 (100%)
Ever spontaneously cleared and re-infected	1 (<1%)
Medical history	
Alcoholism/alcohol abuse	40 (6%)
Illicit substance use	213 (34%)
Depression	3 (<1%)
Malignancy	2 (<1%)
Peripheral neuropathy	12 (2%)
Current	12 (100%)
Diabetes	36 (6%)
Current	36 (100%)
Hyperlipidaemia	19 (3%)
Current	14 (74%)
Recent	2 (11%)
Historic	3 (16%)
Hypertension	65 (10%)
Current	63 (97%)
Historic	2 (3%)
Angina	3 (<1%)
Myocardial infarction	0
Chronic obstructive pulmonary disease	4 (1%)
Tuberculosis	46 (7%)
Recent	3 (7%)
Historic	43 (93%)

	Total
Cigarette smoking status	
Non-smoker	208 (33%)
Past smoker	81 (13%)
Current smoker	335 (54%)

Table S2 (a) DAA exposure

Randomisation	N=621*	Mean (SD) days DAA received (first-line only)	Mean (SD) days DAA received (including any retreatment)	SVR12
SOF/DCV+SOC	77 (12%)	83 (3.4)	83 (3.4)	75/75 (100%)
SOF/DCV+RGT	78 (13%)	53 (16.2)	58 (23.3)	71/76 (93%)
SOF/DCV+I/M	77 (12%)	63 (1.6)	63 (1.6)	75/75 (100%)
SOF/DCV+4w DAA/ IFN	78 (13%)	28 (0.3)	31 (16.0)	73/76 (96%)
SOF/VEL+SOC	76 (12%)	84 (1.1)	86 (12.6)	73/75 (97%)
SOF/VEL+RGT	79 (13%)	53 (18.8)	59 (25.5)	73/79 (92%)
SOF/VEL+I/M	79 (13%)	63 (4.0)	64 (10.4)	76/77 (99%)
SOF/VEL+4w DAA/ IFN	77 (12%)	28 (0.5)	34 (21.5)	70/76 (92%)

* excluding three participants who withdrew immediately after randomisation and did not start treatment (SOF/DCV+SOC, SOF/VEL+RGT, SOF/VEL+4w DAA/IFN).

Table S2 (b) RGT treatment duration

	SOF/DCV	SOF/VEL	Total
	N=78	N=79	N=157
Received 4 weeks	18 (23%)	22 (28%)	40 (25%)
Received 8 weeks	51 (65%)	43 (54%)	94 (60%)
Received 12 weeks	9 (12%)*	14 (18%)	23 (15%)
Median (IQR) HCV VL at Day-7 (IU/mL)	66 (24, 180)	108 (37.2, 250)	93.7 (28.5, 210.5)

*One participant did not attend their Day-7 visit and was allocated 12 weeks treatment.

Table S3 Adherence to first-line therapy (a) by treatment combination (b) by treatment strategy (c) by treatment strategy over time

(a)

	**SOF/DCV**	**SOF/VEL**	**Total**
	N=311	N=313	N=624
Completed correct treatment	292 (94%)	292 (93%)	584 (94%)
LTFU or withdrew consent before EOT	5 (2%)	4 (1%)	9 (1%)
Took less than allocated	14 (5%)	17 (5%)	31 (5%)
Took more than allocated	0	0	0
In those starting first-line	N=310	N=311	N=621
Any missed DAA doses reported	48 (15%)	34 (11%)	82 (13%)
Number of forms reporting missed DAA doses			
0	262 (85%)	277 (89%)	539 (87%)
1	18 (6%)	11 (4%)	29 (5%)
2	16 (5%)	11 (4%)	27 (4%)
3	6 (2%)	6 (2%)	12 (2%)
4	8 (3%)	6 (2%)	14 (2%)
Any missed IFN doses (% of 4w-DAA/IFN)	2 (3%)	7 (9%)	9 (6%)
Any missed DAA or IFN doses	50 (16%)	40 (13%)	90 (14%)
Any weekend DAA doses (% of I/M)	11 (14%)	21 (27%)	32 (21%)

Note: Of those LTFU/withdrew before EOT, three withdrew immediately and took no treatment, six started treatment and remained in follow-up for 7 (3 participants), 4, 3, and 2 weeks. Of those who took less than the prescribed duration, median (IQR) [range] of total treatment taken was 95% (92%, 95%) [88%, 96%]. In all cases this was due to missed doses at the end of treatment.

(b)

	**SOC**	**RGT**	**IM**	**4w DAA/IFN**	**Total**
	N=154	N=158	N=156	N=156	N=624
Completed correct treatment	142 (92%)	157 (99%)	132 (85%)	142 (91%)	584 (94%)
LTFU or withdrew consent before EOT	2 (1%)	1 (1%)	4 (3%)	2 (1%)	9 (1%)
Took less than allocated	10 (6%)	0	20 (13%)	1 (1%)	31 (5%)
Took more than allocated	0	0	0	0	0
In those starting first-line	N=153	N=157	N=156	N=155	N=621
Any missed DAA doses reported*	35 (23%)	18 (11%)	19 (12%)	10 (6%)	82 (13%)
Number of forms reporting missed DAA doses					
0	118 (77%)	139 (89%)	137 (88%)	118 (76%)	539 (87%)
1	9 (6%)	10 (6%)	6 (4%)	4 (3%)	29 (5%)
2	10 (7%)	6 (4%)	7 (4%)	4 (3%)	27 (4%)
3	8 (5%)	2 (1%)	1 (1%)	1 (1%)	12 (2%)
4	8 (5%)	0	5 (3%)	1 (1%)	14 (2%)
Any missed IFN doses	-	-	-	9 (6%)	9 (6%)
Any missed DAA or IFN doses	35 (23%)	18 (11%)	19 (12%)	18 (12%)	90 (14%)
Any weekend DAA doses	-	-	32 (21%)	-	-

* chi-squared p<0.0001. Note: LTFU=lost to follow-up, EOT=end of (first-line) treatment

(c)

	**SOC**	**RGT**	**IM**	**4w DAA/IFN**	**Total**
Day 7		N=154		N=154	N=308
Any missed DAA doses	-	4 (3%)	-	1 (1%)	5 (2%)
Day 14	N=151		N=154	N=155	N=460
Any missed DAA doses	9 (6%)	-	6 (4%)	2 (1%)	17 (4%)
Day 21				N=153	N=153
Any missed DAA doses	-	-	-	6 (4%)	6 (4%)
Day 28	N=153	N=156	N=153	N=151	N=613
Any missed DAA doses	15 (10%)	9 (6%)	9 (6%)	10 (7%)	43 (7%)
Day 56	N=151	N=112	N=151		N=414
Any missed DAA doses	27 (18%)	10 (9%)	14 (9%)	-	51 (12%)
Day 86	N=146	N=22	N=141		N=309
Any missed DAA doses	34 (23%)	5 (23%)	14 (10%)	-	53 (17%)

Note: see **Figure S1** for visit schedule which differed by treatment strategy randomisation.

Table S4 Sensitivity analyses for primary endpoint (a) by treatment combination (b) by treatment strategy

(a)

	**SOF/DCV**	**SOF/VEL**
	N=302	N=307
SVR12	294 (97.4%)	292 (95%)
Primary analysis: Bayesian analysis with 90% CrIs and uninformative priors (**Table 2**)		
90% CrIs, uninformative priors	2.2% (-0.2%, 4.8%) P=0.93 p=0.14	Reference
Secondary analyses: Bayesian analyses		
95% CrIs, uninformative priors	2.2% (-0.8%, 5.3%) P=0.93, p=0.14	Reference
90% CrIs, sceptical priors	0.2% (0.1%, 0.3%) P=0.51, p=0.98	Reference
90% CrIs, enthusiastic priors	0.0% (-0.1%, 0.1%) P=0.51, p=0.98	Reference
Secondary analysis: Frequentist analysis		
95% CIs	2.2% (-0.7%, -5.2%) p=0.14	Reference

(b)

	**SOC**	**RGT**	**IM**	**4w DAA-IFN**
	N=150	N=155	N=152	N=152
SVR12	148 (98.7%)	144 (92.9%)	151 (99.3%)	143 (94.1%)
Primary analysis: Bayesian analysis with 90% CrIs and uninformative priors (**Table 2**)				
90% CrIs, uninformative priors	Reference	-5.7% (-9.6%, -2.3%) P=1.00, p=0.01	0.6% (-1.1%, 2.7%) P=0.26, p=0.51	-4.5% (-8.3%, -1.3%) P=0.99, p=0.02
Secondary analyses: Bayesian analyses				
95% CrIs, uninformative priors	Reference	-5.7% (-10.4%, -1.6%) P=1.00, p=0.01	0.6% (-1.5%, 3.2%) P=0.26, p=0.51	-4.5% (-9.0%, -0.7%) P=0.99, p=0.02
90% CrIs, sceptical priors	Reference	-0.3% (-0.5%, -0.2%) P=1.00, p<0.001	-0.3% (-0.5%, -0.2%) P=1.00, p<0.001	-0.3% (-0.5%, -0.2%) P=1.00, p<0.001
90% CrIs, enthusiastic priors	Reference	-0.0% (-0.0%, 0.0%) P=0.51, p=0.98	0.0% (-0.0%, 0.0%) P=0.49, p=0.99	-0.0% (-0.0%, 0.0%) P=0.50, p=0.99
Secondary analysis: Frequentist analysis				
95% CIs	Reference	-5.8% (-10.2%, -1.3%) p=0.01	0.7% (-1.6%, 2.9%) p=0.55	-4.5% (-8.7%, -0.4%) p=0.03

CrI: credible interval, CI: confidence interval. For Bayesian analyses, P is the probability that the treatment effect is <0%, i.e. that the test is worse than reference, and p is an approximation of a two-sided frequentist p-value (here double the probability that the treatment effect is >0).

Table S5 SVR12 by factorial randomisation

Strategy randomisation	SOF/DCV	SOF/VEL	Total	Risk difference (95% CI)
SOC	75/75 (100%)	73/75 (97.3%)	148/150 (98.7%)	-
RGT	71/76 (93.4%)	73/79 (92.4%)	144/155 (92.9%)	1.1% (-7.0%, +9.1%)
IM	75/75 (100%)	76/77 (98.7%)	151/152 (99.3%)	-
4w DAA/IFN	73/76 (96.1%)	70/76 (92.1%)	143/152 (94.1%)	+3.9% (-3.5%. +11.4%)

Regimen randomisation	SOC	RGT	IM	4w DAA/IFN	Total
SOF/DCV	75/75 (100%)	71/76 (93.4%)	75/75 (100%)	73/76 (96.1%)	294/302 (97%)
SOF/VEL	73/75 (97.3%)	73/79 (92.4%)	76/77 (98.7%)	70/76 (92.1%)	292/307 (95%)
Risk difference (95% CI)	Reference	-5.0% (-11.9%,+1.9%)	1.4% (-3.1%,+5.8%)	-5.2% (-12.2%,+1.9%)	

Note: risk differences not estimable using Bayesian non-informative priors where no failures in one group. Using penalised maximum likelihood logistic regression, heterogeneity p=0.79.

Table S6 Subgroup analyses for primary endpoint (a) by treatment combination (b) by treatment strategy

	SOF/DCV	SOF/VEL	Total	Risk difference SOF/DCV – SOF/VEL (95% CI)
Baseline HCV VL				
>=6,000,000 IU/ml	87/88 (99%)	83/89 (93%)	170/177 (96%)	+5.5% (-0.0%,+10.9%)
<6,000,000 IU/ml	207/214 (97%)	209/218 (96%)	416/432 (96%)	+0.9% (-2.6%,+4.4%)
				Heterogeneity p-value=0.194
Fractional polynomial for interaction with continuous variable				Heterogeneity p-value=0.600
HCV genotype: G6 vs non-G6				
non-G6	153/159 (96%)	152/162 (94%)	305/321 (95%)	+2.4% (-2.3%,+7.1%)
G6	141/143 (99%)	140/145 (97%)	281/288 (98%)	+2.0% (-1.5%,+5.5%)
				Heterogeneity p-value=0.701
HCV genotype: G6 vs G1 vs G2 vs G3				
G1	119/122 (98%)	121/128 (95%)	240/250 (96%)	+3.2% (+1.5%,+8.0%)
G2	25/28 (89%)	18/21 (86%)	43/49 (88%)	+6.0% (-12.8%,+24.7%)
G3	9/9 (100%)	13/13 (100%)	22/22 (100%)	-
G6	141/143 (99%)	140/145 (97%)	281/288 (98%)	+2.0% (-1.5%,+5.5%)
				Heterogeneity p-value=0.954
Age				
First tercile	107/111 (96%)	105/110 (95%)	212/221 (96%)	+1.2% (-3.9%,+6.4%)
Second tercile	92/93 (99%)	103/110 (94%)	195/203 (96%)	+5.1% (+0.2%,+10.0%)
Third tercile	95/98 (97%)	84/87 (97%)	179/185 (97%)	+0.2% (-4.9%,+5.3%)
				Heterogeneity p-value=0.406
Fractional polynomial for interaction with continuous variable				Heterogeneity p-value=0.733
Sex				
Male	223/229 (97%)	218/229 (95%)	441/458 (96%)	+2.3% (-1.1%,+5.7%)
Female	71/73 (97%)	74/78 (95%)	145/151 (96%)	+2.0% (-4.1%,+8.1%)
				Heterogeneity p-value=0.909
BMI				
First tercile	100/102 (98%)	101/107 (94%)	201/209 (96%)	+4.1% (-1.1%,+9.2%)
Second tercile	96/99 (97%)	96/101 (95%)	192/200 (96%)	+1.6% (-3.7%,+6.9%)
Third tercile	98/101 (97%)	95/99 (96%)	193/200 (96%)	+1.0% (-4.1%,+6.0%)
				Heterogeneity p-value=0.677
Fractional polynomial for interaction with continuous variable				Heterogeneity p-value=0.333
HIV coinfection				
HIV uninfected	255/262 (97%)	253/266 (95%)	508/528 (96%)	+2.3% (-0.9%,+5.5%)
HIV infected	39/40 (98%)	39/41 (95%)	78/81 (96%)	+2.1% (-5.9%,+10.2%)
				Heterogeneity p-value=0.992
Treatment response at D14 (non-RGT groups only)				
<LLOQ	92/94 (98%)	90/94 (96%)	182/188 (97%)	+1.2% (-3.6%,+6.0%)
>=LLOQ	128/129 (99%)	128/133 (96%)	256/262 (98%)	+3.3% (-0.3%,+7.0%)
				Heterogeneity p-value=0.336
Study centre				
HCMC	160/165 (97%)	157/165 (95%)	317/330 (96%)	+1.8% (-2.4%,+5.9%)
Hanoi	134/137 (98%)	135/142 (95%)	269/279 (96%)	+2.8% (-1.5%,+7.1%)
				Heterogeneity p-value=0.667
IFNL4 genotype				
CC	251/257 (98%)	255/266 (96%)	506/523 (97%)	+1.8% (-1.2%, +4.8%)
CT/TT	43/45 (96%)	37/41 (90%)	80/86 (93%)	+5.2% (-5.3%, +15.8%)
				Heterogeneity p-value=0.799
Illicit substance use				
No	194/199 (97%)	196/205 (96%)	390/404 (97%)	+1.8% (-1.7%, +5.3%)
Yes	100/103 (97%)	96/102 (94%)	196/205 (96%)	+3.2% (-2.4%, +8.8%)
				Heterogeneity p-value=0.787
Fibroscan result				
<5kPa	71/73 (97%)	54/59 (92%)	125/132 (95%)	+5.6% (+2.3%, +13.4%)
5-7kPa	141/143 (99%)	144/150 (96%)	285/293 (97%)	+2.6% (-1.1%, +6.3%)
>=7kPa	82/86 (95%)	94/98 (96%)	176/184 (96%)	-0.6% (-6.4%, +5.3%)
				Heterogeneity p-value=0.400

	SOF/DCV	SOF/VEL	Total	Risk difference SOF/DCV – SOF/VEL (95% CI)
Fractional polynomial for interaction with continuous variable				Heterogeneity p-value=0.219
Missed DAA doses				
No missed doses	246/254 (97%)	261/274 (95%)	507/528 (96%)	-
Missed doses	48/48 (100%)	30/32 (94%)	78/80 (98%)	-
Missed visits				
No missed visits	279/286 (98%)	277/288 (96%)	556/574 (97%)	+1.5% (-1.3%, +4.4%)
Any missed visits	15/16 (94%)	15/19 (79%)	30/35 (86%)	+3.0% (-22.1%, +28.0%)
				Heterogeneity p-value=0.86
Time of enrolment				
Enrolled pre-COVID-19 lockdowns	75/78 (96%)	77/80 (96%)	152/158 (96%)	-0.2% (-6.1%, +5.7%)
Enrolled between lockdowns and 01/06/2022	84/87 (97%)	84/86 (98%)	168/173 (97%)	-1.2% (-6.1%, +3.8%)
Enrolled after 01/06/2022	135/137 (99%)	131/141 (93%)	266/278 (96%)	+5.7% (+1.1%, +10.3%)
				Heterogeneity p-value=0.16

(b)

	SOC	RGT	I/M	PEG-IFN	Total
Baseline HCV VL					
>=6,000,000 IU/ml	33/34 (97%)	43/43 (100%)	54/55 (98%)	40/45 (89%)	170/177 (96%)
Risk difference (95% CI)	Reference	-	1.3% (-5.6%, 8.2%)	-7.9% (-18.6%, 2.9%)	
<6,000,000 IU/ml	115/116 (99%)	101/112 (90%)	97/97 (100%)	103/107 (96%)	416/432 (96%)
Risk difference (95% CI)	Reference	-9.1% (-14.8%, -3.3%)	-	-2.9% (-6.8%, 1.1%)	
Fractional polynomial for interaction with continuous variable					Heterogeneity p-value=0.271
HCV genotype: G6 vs non-G6					
non-G6	78/78 (100%)	75/81 (93%)	79/80 (99%)	73/82 (89%)	305/321 (95%)
G6	70/72 (97%)	69/74 (93%)	72/72 (100%)	70/70 (100%)	281/288 (98%)
Risk difference (95% CI)	Reference	-3.9% (-10.8%, 2.9%)	-	-	
HCV genotype: G6 vs G1 vs G2 vs G3					
G1	59/59 (100%)	62/66 (94%)	59/60 (98%)	60/65 (92%)	240/250 (96%)
G2	11/11 (100%)	9/11 (82%)	14/14 (100%)	9/13 (69%)	43/49 (88%)
G3	8/8 (100%)	4/4 (100%)	6/6 (100%)	4/4 (100%)	22/22 (100%)
G6	70/72 (97%)	69/74 (93%)	72/72 (100%)	70/70 (100%)	281/288 (98%)
Risk difference (95% CI)	Reference	-3.9% (-10.7%, 2.9%)	-	-	
Age					
First tercile	54/56 (96%)	59/63 (94%)	56/57 (98%)	43/45 (96%)	212/221 (96%)
Risk difference (95% CI)	Reference	-2.9% (-10.6%, 4.8%)	1.7% (-4.1%, 7.5%)	-1.0% (-8.7%, 6.7%)	
Second tercile	52/52 (100%)	39/43 (91%)	54/54 (100%)	50/54 (93%)	195/203 (96%)
Third tercile	42/42 (100%)	46/49 (94%)	41/41 (100%)	50/53 (94%)	179/185 (97%)
Fractional polynomial for interaction with continuous variable					Heterogeneity p-value=0.377
Sex					
Male	110/112 (98%)	106/115 (92%)	115/116 (99%)	110/115 (96%)	441/458 (96%)
Risk difference (95% CI)	Reference	-6.1% (-11.7%, -0.6%)	1.0% (-2.0%, 4.0%)	-2.4% (-6.8%, 2.0%)	
Female	38/38 (100%)	38/40 (95%)	36/36 (100%)	33/37 (89%)	145/151 (96%)
BMI					
First tercile	54/55 (98%)	54/59 (92%)	53/53 (100%)	40/42 (95%)	201/209 (96%)
Risk difference (95% CI)	Reference	-6.8% (-14.7%, 1.1%)	-	-3.2% (-10.6%, 4.3%)	
Second tercile	43/44 (98%)	47/50 (94%)	47/47 (100%)	55/59 (93%)	192/200 (96%)
Risk difference (95% CI)	Reference	-3.3% (-11.2%, 4.6%)	-	-4.2% (-12.0%, 3.7%)	
Third tercile	51/51 (100%)	43/46 (93%)	51/52 (98%)	48/51 (94%)	193/200 (96%)
Fractional polynomial for interaction with continuous variable					Heterogeneity p-value=0.927
HIV coinfection					
HIV uninfected	127/129 (98%)	125/134 (93%)	129/130 (99%)	127/135 (94%)	508/528 (96%)
Risk difference (95% CI)	Reference	-5.2% (-10.0%, -0.5%)	0.8% (-1.8%, 3.4%)	-4.3% (-8.7%, 0.2%)	
HIV infected	21/21 (100%)	19/21 (90%)	22/22 (100%)	16/17 (94%)	78/81 (96%)
Treatment response at D14 (non-RGT arms only)					
<LLOQ	60/61 (98%)	-	85/86 (99%)	37/41 (90%)	182/188 (97%)
Risk difference (95% CI)	Reference	-	0.3% (-3.6%, 4.1%)	-8.0% (-17.1%, 1.2%)	
>=LLOQ	87/88 (99%)	-	66/66 (100%)	103/108 (95%)	256/262 (98%)
Risk difference (95% CI)	Reference	-	-	-3.3% (-7.7%, 1.2%)	
Study centre					
HCMC	81/81 (100%)	78/83 (94%)	83/83 (100%)	75/83 (90%)	317/330 (96%)
Hanoi	67/69 (97%)	66/72 (92%)	68/69 (99%)	68/69 (99%)	269/279 (96%)
Risk difference (95% CI)	Reference	-5.4% (-12.9%, 2.0%)	1.5% (-3.4%, 6.3%)	1.5% (-3.4%, 6.3%)	

	SOC	RGT	I/M	PEG-IFN	Total
IFNL4 genotype					
CC	133/134 (99%)	126/134 (94%)	125/126 (99%)	122/129 (95%)	506/523 (97%)
Risk difference (95% CI)	Reference	-5.2% (-9.4%, -1.0%)	-0.1% (-2.2%, +2.1%)	-4.7% (-8.9%, -0.5%)	
CT/TT	15/16 (94%)	18/21 (86%)	26/26 (100%)	21/23 (91%)	80/86 (93%)
Risk difference (95% CI)	Reference	-7.5% (-27.2%,+12.1%)	-	-1.2% (-18.1%, +15.6%)	
Illicit substance abuse					
No	97/97 (100%)	96/104 (92%)	97/97 (100%)	100/106 (94%)	390/404 (97%)
Yes	51/53 (96%)	48/51 (94%)	54/55 (98%)	43/46 (93%)	196/205 (96%)
Risk difference (95% CI)	Reference	-2.2% (-10.5%,+6.1%)	+2.0% (-4.2%,+8.2%)	-2.7% (-11.5%,+6.0%)	
Fibroscan result					
<5kPa	26/27 (96%)	36/40 (90%)	30/30 (100%)	33/35 (94%)	125/132 (95%)
Risk difference (95% CI)	Reference	-7.3% (-19.4%,+4.8%)	-	-1.8% (--11.8%,+8.2%)	
5-7kPa	76/77 (99%)	60/64 (94%)	76/77 (99%)	73/75 (97%)	285/293 (97%)
Risk difference (95% CI)	Reference	-4.5% (-10.6%,+1.6%)	+0.1% (-3.4%, +3.6%)	-1.6% (-6.5%,+3.1%)	
>=7kPa	46/46 (100%)	48/51 (94%)	45/45 (100%)	37/42 (88%)	176/184 (96%)
Fractional polynomial for interaction with continuous variable					Heterogeneity p-value=0.486
Missed DAA doses					
No missed doses	115/116 (99%)	125/136 (92%)	133/134 (99%)	134/142 (94%)	507/528 (96%)
Risk difference (95% CI)	Reference	-7.2% (-12.0%, -2.3%)	+0.1% (-2.1%, 2.4%)	-4.7% (-8.8%, -0.6%)	
Missed doses	33/34 (97%)	18/18 (100%)	18/18 (100%)	9/10 (90%)	78/80 (98%)
Risk difference (95% CI)	Reference	-	-	-7.6% (-27.7%, 12.4%)	
Missed visits					
No missed visits	141/143 (99%)	138/147 (94%)	138/139 (99%)	139/145 (96%)	
Risk difference (95% CI)	Reference	-4.7% (-9.0%, -0.4%)	+0.7% (-1.7%, +3.1%)	-2.8% (-6.6%, +1.0%)	
Any missed visits	7/7 (100%)	6/8 (75%)	13/13 (100%)	4/7 (57%)	30/35 (86%)
Time of enrolment					
Enrolled pre-COVID-19 lockdowns	41/41 (100%)	36/39 (92%)	40/40 (100%)	35/38 (92%)	152/158 (96%)
Enrolled between lockdowns and 01/06/2022	40/40 (100%)	42/43 (98%)	43/43 (100%)	43/47 (91%)	168/173 (97%)
Enrolled after 01/06/2022	67/69 (97%)	66/73 (90%)	68/69 (99%)	65/67 (97%)	266/278 (96%)
Risk difference (95% CI)	Reference	-6.8% (-14.6%, +1.0%)	+1.4% (-3.4%, 6.2%)	-0.1% (-5.8%, +5.5%)	

Note: 95% CI not estimable where no failures in either the reference or any specific experimental arm. In this scenario no risk difference is shown. Heterogeneity p-values shown where models would converge (not always possible where some subgroups had 100% cure rates even using penalised regression). Subgroup analyses were prespecified in the protocol with the exception of illicit substance use and Fibroscan result (added by investigators); and calendar time (divided into periods before the first regional lockdown in each site (HCMC 21 May 2021, Hanoi 11 June 2021), up to 1 June 2022 and subsequently), missing first-line visits and missing any DAA doses, added at the request of a reviewer, see **Supplementary Methods**.

Table S7 SVR12 by duration of treatment in RGT

Treatment length	SVR12
4 weeks	31/39 (79.5%)
8 weeks	91/93 (97.9%)
12 weeks	22/23 (95.7%)

Table S8 Association between SVR12 and genotype

	Primary analysis (Table S4)	Adjusted analysis: Genotype 6 vs non-6	Adjusted analysis: Genotype 1+3* vs 2 vs 6	Adjusted analysis: Genotype 6 vs non-6 with IFN4L	Adjusted analysis: Genotype 1+3* vs 2 vs 6 with IFN4L
Regimen: SOF/DCV vs SOF/VEL	2.2% (-0.8%, 5.3%) P=0.14	2.3% (-0.6, 5.3) P=0.12	2.7% (-0.1, 5.8) P=0.058	2.3% (-0.5, 5.3) P=0.11	2.7% (-0.2, 5.6) P=0.065
Strategy: RGT vs SoC	-5.7% (-10.4%, -1.6%) P=0.01	-5.8% (-10.5, -1.7) P=0.01	-5.7% (-10.4, -1.6) P=0.01	-5.8% (-10.5, -1.7) P=0.01	-5.6% (-10.3, -1.5) P=0.01
I/M vs SoC	0.6% (-1.5%, 3.2%) P=0.51	0.6% (-1.4, 3.2) P=0.49	0.8% (-1.1, 3.7) P=0.36	0.7% (-1.4, 3.4) P=0.46	0.9% (-1.0, 3.9) P=0.33
IFN vs SoC	-4.5% (-9.0%, -0.7%) P=0.02	-4.4% (-8.8, -0.7) P=0.02	-4.4% (-8.8, -0.4) P=0.03	-4.3% (-8.7, -0.4) P=0.03	-4.3% (-8.9, -0.3) P=0.04
Genotype:	6 vs non-6: 2.5% (-0.3, 5.5) P=0.09	6 vs non-6: 2.6% (-0.3, 5.7) P=0.08	6 vs 1+3*: 1.0% (-1.6, 3.8) P=0.44	6 vs non-6: 2.6% (-0.3, 5.7) P=0.08	6 vs 1+3*: 1.0% (-1.6, 3.9) P=0.44
			2 vs 1+3*: -15.8% (-30.7, -4.2) P=0.003		2 vs 1+3*: -15.0% (-30.4, -3.8) P=0.003
Site: Hanoi vs HCMC	0.1% (-2.9, 3.1) P=0.94	0.03% (-3.1, 3.1) P=0.99	-1.1% (-4.7, 2.1) P=0.49	0.01% (-3.0, 2.9) P=0.99	-1.1% (-4.6, 2.1) P=0.50
Illicit substance use		-1.4% (-5.3, 1.8) P=0.41	-2.2% (-6.2, 1.2) P=0.22	-1.7% (-5.6, 1.6) P=0.35	-2.3% (-6.4, 1.1) P=0.19
Age per 7 years older		-0.3% (-1.4, 0.8) P=0.64	0.1% (-1.0, 1.3) P=0.86	-0.3% (-1.4, 0.9) P=0.64	0.1% (-1.0, 1.3) P=0.85
BMI per 2.1 kg/m^2 higher		-0.2% (-1.3, 0.8) P=0.63	-0.1% (-1.1, 1.0) P=0.87	-0.2% (-1.3, 0.8) P=0.64	-0.0% (-1.1, 1.0) P=0.94
VL (log_{10}) per 0.6log10 higher		-0.5% (-1.6, 0.4) P=0.28	-0.7% (-1.8, 0.2) P=0.14	-0.5% (-1.6, 0.4) P=0.25	-0.7% (-1.9, 0.2) P=0.13
IFN4L: CC vs CT/TT				3.9% (-0.5, 10.4) P=0.10	3.5% (-0.7, 9.7) P=0.12

* 100% SVR12 in genotype 3 therefore combined with genotype 1 in analysis.

Note: showing point estimates and 95% CrIs. P is an approximation of a two-sided frequentist p-value, showing the two-sided probability that the difference vs reference is not zero from the Bayesian model. Increments for continuous factors are approximately half the width of the interquartile range. Using an equivalent frequentist model, the frequentist heterogeneity p-value for genotype by regimen heterogeneity was 0.70 for the primary analysis (G6 vs non-6 adjusted only for site **Table S6**) and p=0.65 for 6 vs 1+3 vs 2.

Table S9 Details of SVR12 failures (a) overall (b) by participant
(a)

	SOF/DCV + SOC	SOF/DCV + RGT	SOF/DCV + I/M	SOF/DCV + PEG-IFN	SOF/VEL + SOC	SOF/VEL + RGT	SOF/VEL + I/M	SOF/VEL + PEG-IFN	Total
Meeting failure criteria	0/75	5/76 (7%)	0/75	3/76 (4%)	2/75 (3%)	6/79 (8%)	1/77 (1%)	6/76 (8%)	23/609 (4%)
**Of failures**									
Failed at EOT+4	0/0	4/5	0/0	1/3	0/2	5/6	1/1	1/6	12/23 (52%)
Failed at EOT+8	0/0	0/5	0/0	2/3	2/2	1/6	0/1	4/6	9/23 (39%)
Failed at EOT+12	0/0	1/5	0/0	0/3	0/2	0/6	0/1	0/6	1/23 (4%)
Failed past EOT+12	0/0	0/5	0/0	0/3	0/2	0/6	0/1	1*/6	1/23 (4%)
Rebound	0/0	5/5	0/0	2/3	2/2	6/6	1/1	4/6	20/23 (87%)
Lack of response	0/0	0/5	0/0	1/3	0/2	0/6	0/1	2/6	3/23 (13%)
Median (IQR) HCV VL at failure	-	75399 (10334, 1950000)	-	8120000 (47098, 8289174)	(49900, 3390000)	173991 (5600, 308000)	1680	7294307 (382636, 11000000)	308000 (47098, 8120000)
**Of all participants**									
>LLOQ VL at EOT	0/1	0/0	0/0	0/4	0/0	0/1	0/1	0/4	0/11
LLOD-LLOQ VL at EOT	0/7	0/5	0/11	1/14 (7%)	0/7	1/11 (9%)	0/4	2**/15 (13%)	4/74 (5%)
<LLOD VL at EOT	0/59	5/66 (8%)	0/54	2/57 (4%)	2/57 (4%)	5/64 (8%)	1/65 (2%)	2**/52 (4%)	17/474 (4%)*
No missed doses	0/56	5/64 (8%)	0/65	3/67 (4%)	1/60 (2%)	6/73 (8%)	1/69 (1%)	2/67 (3%)	18/521 (3%)
Missed DAA	0/19	0/12	0/10	0/7	1/15 (7%)	0/6	0/8	0/2	1/79 (1%)
Missed PEG-IFN	N/A	N/A	N/A	0/2	N/A	N/A	N/A	4/7 (57%)†	4/9 (44%)
Subtype 1A	0/15	2/18 (11%)	0/17	1/14	0/20	2/23 (9%)	1/19 (5%)	2/19 (11%)	8/145 (6%)
Subtype 1B	0/13	0/16	0/11	0/17	0/10	0/9	0/13	2/15 (13%)	2/104 (2%)
Subtype 2A	0/7	1/5 (20%)	0/4	1/5 (20%)	0/2	1/3 (33%)	0/3	2/4 (50%)	5/33 (15%)
Subtype 2 other	0/0	0/1	0/2	1/4 (25%)	0/2	0/2	0/5	0/0	1/16 (6%)
Subtypes 3A/B	0/3	0/0	0/5	0/1	0/5	0/4	0/1	0/3	0/22
Subtype 6A	0/26	1/14 (7%)	0/24	0/22	1/23 (4%)	3/31 (10%)	0/19	0/15	5/174 (3%)
Subtype 6 other	0/10	1/22 (5%)	0/12	0/13	1/13 (8%)	0/7	0/17	0/20	2/114 (2%)

*This participant failed 30 weeks past their EOT visit (they did not attend any visits between EOT+4 and the time of treatment failure).

**Two participants with treatment failure had no recorded EOT VL (both SOF/VEL+IFN). At the visit prior to their scheduled EOT visit, both participant's VLs were <LLOD.

† One participant reported both missed DAAs and PEG-IFN, see (b) below.

Note: LLOD=lower limit of detection, LLOQ=lower limit of quantification.

(b)

Trial number	Regimen	Strategy	Treatment length (weeks)	Age (years)	Sex	Geno-type	BMI (kg/m^2)	Fibroscan result (kPa)	Enrolment VL (IU/ml)	VL at decision time point (IU/ml)	Missed study drug
RGT 4 week – n=8/23											
	VEL	RGT	4	42	Male	1A	19.5	5.6	73833	LLOD-LLOQ	None
	VEL	RGT	4	43	Female	2A	25.7	3.7	225000	<LLOD	None
	VEL	RGT	4	66	Male	6A	20.4	6.8	393063	LLOD-LLOQ	None
	DCV	RGT	4	36	Male	6NA	23.1	3.8	417804	LLOD-LLOQ	None
	DCV	RGT	4	63	Male	1A	18.9	7.9	478000	LLOD-LLOQ	None
	DCV	RGT	4	39	Male	6A	19.7	6.1	1150000	LLOD-LLOQ	None
	VEL	RGT	4	44	Female	1A	21.8	5.4	2530000	<LLOD	None
	VEL	RGT	4	54	Male	6A	25.7	4.8	2650000	LLOD-LLOQ	None
IFN missed doses – n=4/23											
	VEL	IFN	4	46	Male	2A	27.6	7.1	2210000	N/A	1 IFN dose
	VEL	IFN	4	54	Female	2A	19.1	4.4	3450000	N/A	1 IFN dose
	VEL	IFN	4	46	Male	1B	21.0	5.8	7932196	N/A	1 IFN dose
	VEL	IFN	4	30	Male	1B	15.3	7.0	25428032	N/A	7% of DAA, 2 IFN doses
Other – n=11/23											
	VEL	SOC	12	33	Male	6E	20.7	3.4	74600	N/A	None
	VEL	SOC	12	32	Male	6A	21.8	5.5	19200000	N/A	7% of DAA*
	DCV	RGT	8	18	Male	1A	22.3	7.0	529000	17.2	None
	DCV	RGT	8	48	Male	2A	24.2	4.7	2890000	45.4	None
	VEL	RGT	12	37	Male	6A	19.9	8.8	5570000	421	None
	VEL	IM	12	39	Male	1A	29.8	5.1	6820000	N/A	None
	DCV	IFN	4	52	Female	2NA	29.5	N/A [FIB-4=1.3]	6410000	N/A	None
	DCV	IFN	4	70	Male	2A	22.3	8.1	1550234	N/A	None
	DCV	IFN	4	39	Female	1A	27.5	5.5	1817816	N/A	None
	VEL	IFN	4	48	Male	1A	21.1	7.5	7810000	N/A	None
	VEL	IFN	4	41	Female	1A	22.9	2.9	18258272	N/A	None

* missed 2 doses D14-D28; 2 doses D28-D56

Table S10 Serious adverse events

Event	SOF/DCV + SOC	SOF/DCV + RGT	SOF/DCV + IM	SOF/DCV + DAA/IFN	SOF/VEL + SOC	SOF/VEL + RGT	SOF/VEL + IM	SOF/VEL + DAA/IFN	Total
death with unknown cause	0	0	0	1	0	0	0	0	1
leukopenia	0	0	0	0	0	0	0	1	1
sepsis	0	0	0	0	0	0	1	0	1
MRSA bacteremia and skin and soft tissue infection	0	0	0	0	0	1	0	0	1
cough	0	0	0	0	0	1	0	0	1
pneumonia	0	1	0	0	0	0	0	0	1
pulmonary tuberculosis	0	0	0	0	0	1	0	0	1
acute diverticulitis	1	0	0	0	0	0	0	0	1
gastroenteritis with hypovolemia and acute kidney injury	0	1	0	0	0	0	0	0	1
gastrointestinal infection	0	0	0	0	1	0	0	0	1
other bacterial intestinal infections	0	0	0	0	0	0	0	1	1
fracture of other and unspecified metacarpal bone; open wound of wrist and hand	0	0	0	0	0	0	0	1	1
injury of left achilles tendon	0	0	0	0	1	0	0	0	1
condyle humerus fracture	0	0	0	0	1	0	0	0	1
open rupture of distal phalanx in left thumb and rupture of left thumb extensor tendon	0	0	1	0	0	0	0	0	1
hospitalization for drug withdrawal	0	0	0	0	0	0	0	1	1
pain in right ankle	0	0	0	0	0	1	0	0	1
calculus of ureter	0	0	0	0	0	0	1	0	1
recurrent bile duct stones	0	1	0	0	0	0	0	0	1

Table S11 Grade 3/4 AEs

Event	SOF/DCV + OC	SOF/DCV + RGT	SOF/DCV + IM	SOF/DCV + DAA-IFN	SOF/VEL + SOC	SOF/VEL + RGT	SOF/VEL + IM	SOF/VEL + DAA-IFN	Total
anemia	0	0	0	0	0	1	0	0	1
leukopenia	0	0	0	0	0	0	0	1	1
thrombocytopenia	0	0	0	0	0	0	0	1	1
positive sputum smear with *M. tuberculosis*	0	0	0	0	0	0	0	1	1
subcutaneous abscess of left chest	0	0	0	0	0	0	1	0	1

Table S12 SAEs and grade 3/4 AEs considered probably or possibly related to any study drug

Regimen	Strategy	Event	Type of event	Grade	Relatedness to DAA	Relatedness to DAA-IFN
SOF/VEL	DAA-IFN	other bacterial intestinal infections	SAE	3	Not related	Probably
SOF/VEL	DAA-IFN	leukopenia	SAE	1	Not related	Probably
SOF/VEL	DAA-IFN	thrombocytopenia	AE	3	Not related	Probably
SOF/VEL	DAA-IFN	positive sputum smear with *M. tuberculosis*	AE	3	Unlikely	Possibly

Table S13 Adverse reactions (possibly/probably/definitely related to trial drugs)

Event	SOF/DCV + SOC	SOF/DCV + RGT	SOF/DCV + I/M	SOF/DCV + PEG-IFN	SOF/VEL + SOC	SOF/VEL + RGT	SOF/VEL + I/M	SOF/VEL + PEG-IFN	Total
anorexia	0	0	0	1	0	0	0	1	2
arthralgia	0	0	0	1	0	0	1	1	3
blurred vision	0	0	0	0	0	0	0	1	1
diarrhea	1	0	0	2	1	0	0	0	4
dizziness	0	0	1	1	0	0	2	0	4
fatigue	1	0	0	3	0	0	1	5	10
fever	0	0	0	16	0	0	0	23	39
flu-like symptoms	0	0	0	8	0	0	0	2	10
gastritis	0	1	0	0	0	0	0	1	2
GI disorder	0	0	0	1	0	0	0	0	1
headache	0	1	1	1	0	0	0	1	4
hemoptysis/ suspected pulmonary tuberculosis	0	0	0	0	0	0	0	2	2
injection site reactions	0	0	0	1	0	0	0	1	2
insomnia	0	0	1	1	1	0	0	1	4
myalgia	0	0	0	2	0	0	0	0	2
nausea	1	2	0	0	0	1	0	0	4
rash	0	0	0	1	0	0	0	1	2
tinnitus	0	0	0	1	0	0	0	0	1
vomitting	0	0	0	1	0	0	0	0	1
hct, wbc, plt decreased	0	0	0	1	0	0	0	0	1
neutropenia	0	0	0	1	0	0	0	5	6
leukopenia	0	0	0	10	0	0	0	8	18
thrombocytopenia	0	0	0	15	0	0	0	20	35
thrombocytopenia + leukopenia	0	0	0	7	0	0	0	6	13
thrombocytopenia + neutropenia	0	0	0	1	0	0	0	1	2
decreased TSH	0	0	0	1	0	0	0	0	1
tsh out of normal range (low then high)	0	0	0	1	0	0	0	0	1
other bacterial intestinal infections	0	0	0	0	0	0	0	1	1
positive sputum smear with mtb	0	0	0	0	0	0	0	1	1

Table S14 Duration and grade of adverse reactions (ARs) (possibly/probably/definitely related to trial drugs)

	SOC	RGT	IM	DAA-IFN	Total
Number of ARs	N=5	N=5	N=7	N=155	N=172
Median (IQR) length of ARs (days)	5 (4, 7)	5 (2, 9)	30 (3, 57)	28 (2, 84)	28 (2, 83)
Grade of AR					
1	5 (100%)	5 (100%)	6 (86%)	123 (77%)	139 (79%)
2	0	0	1 (14%)	34 (21%)	35 (20%)
3	0	0	0	3 (2%)	3 (2%)
Number of total reportable AEs*	N=10	N=13	N=13	N=163	N=199
Grade of event:					
1	6 (60%)	8 (53%)	7 (54%)	126 (75%)	147 (71%)
2	0	0	2 (15%)	36 (21%)	38 (18%)
3	2 (20%)	6 (40%)	3 (23%)	5 (3%)	16 (8%)
4	0	1 (7%)	1 (8%)	0	4 (2%)
5	0	0	0	1 (1%)	1 (<1%)

* SAEs, Grade 3/4 AEs, AEs related to or causing change in trial drugs.

Note: 5 ARs associated with IFN and 4 other events (3 in the RGT arm, 1 in the I/M arm) were ongoing when the participant exited the trial so resolution date and length of event are unknown (counted to end of trial follow-up).

Table S15 Duration and grade of specific adverse reactions (possibly/probably/definitely related to trial drugs) in the interferon arm

	SOF/DCV+DAA/IFN	SOF/VEL+DAA/IFN	DAA/INF Total
Flu-like symptoms	N=30	N=31	N=61
Median (IQR) length of total events (days)	2 (1, 2)	2 (1, 2)	2 (1, 2)
Time of onset (median (QR) weeks since randomisation)	1 (1, 2)	1 (1, 2)	1 (1, 2)
Grade of event:			
1	26 (87%)	21 (68%)	47 (77%)
2	4 (13%)	10 (32%)	14 (23%)
Thrombocytopenia*	N=23	N=27	N=50
Median (IQR) length of total events (days)*	83 (28, 84)	84 (61, 84)	84 (56, 84)
Grade of event:			
1	23 (100%)	25 (93%)	48 (96%)
2	0	1 (4%)	1 (2%)
3	0	1 (4%)	1 (2%)
Leukopenia*	N=17	N=14	N=31
Median (IQR) length of total events (days)*	83 (28, 84)	84 (56, 85)	84 (56, 84)
Grade of event:			
1	12 (71%)	9 (64%)	21 (68%)
2	5 (29%)	5 (36%)	10 (32%)
Neutropenia*	N=2	N=6	N=8
Median (IQR) length of total events (days)*	(28, 28)	56 (28, 56)	42 (28, 56)
Grade of event:			
1	1 (50%)	6 (100%)	7 (88%)
2	1 (50%)	0	1 (13%)

* Full blood count only required at EOT and EOT+12 and therefore duration depended on when clinicians chose to repeat blood tests; see Table S16 for prevalence of abnormalities at these timepoints.

Note: Flu-like symptoms includes any event reported as flu-like symptoms, fever, myalgia, fatigue or headache. Additionally, 4 participants in the non-IFN groups reported flu-like symptoms (fatigue or headache) related to study drug (1 standard, 1 RGT, 1 I/M; all grade 1; median (IQR) length 8 days (5, 20)) and one participant had a Grade 1 thrombocytopenia that had not resolved by EOT+12

Table S16 Adverse reactions causing change to trial drugs

Event	SOF/DCV + SOC	SOF/DCV + RGT	SOF/DCV + IM	SOF/DCV + DAA-IFN	SOF/VEL + SOC	SOF/VEL + RGT	SOF/VEL + IM	SOF/VEL + DAA-IFN	Total
hemoptysis/ suspected pulmonary tuberculosis	0	0	0	0	0	0	0	2	2
other bacterial intestinal infections	0	0	0	0	0	0	0	1	1
positive sputum smear with *M. tuberculosis*	0	0	0	0	0	0	0	1	1
SARS-CoV-2 infection	0	0	0	0	0	0	0	1	1

Table S17 Haematology test results

	EOT					EOT+12				
	SOC	RGT	IM	DAA/IFN	Total	SOC	RGT	IM	DAAIFN	Total
Anaemia	N=138	N=145	N=132	N=149	N=564	N=133	N=129	N=136	N=125	N=523
Grade 0	119 (86%)	115 (79%)	104 (79%)	116 (78%)	454 (81%)	110 (83%)	103 (80%)	104 (76%)	97 (78%)	414 (79%)
Grade 1	18 (13%)	29 (20%)	27 (10%)	33 (22%)	107 (19%)	23 (17%)	25 (19%)	31 (23%)	27 (22%)	106 (20%)
Grade 2	1 (1%)	1 (1%)	1 (1%)	0	3 (1%)	0	1 (1%)	1 (1%)	1 (1%)	3 (1%)
Leukopenia	N=138	N=145	N=132	N=149	N=564	N=133	N=129	N=136	N=125	N=523
Grade 0	134 (97%)	140 (97%)	124 (94%)	134 (90%)	465 (82%)	132 (99%)	122 (95%)	130 (96%)	122 (98%)	506 (97%)
Grade 1	4 (3%)	5 (3%)	8 (6%)	15 (10%)	84 (15%)	1 (1%)	7 (5%)	5 (4%)	3 (2%)	16 (3%)
Grade 2	0	0	0	0	15 (3%)	0	0	1 (1%)	0	1 (0.2%)
Thrombocytopenia	N=138	N=145	N=132	N=149	N=564	N=133	N=129	N=136	N=125	N=523
Grade 0	138 (100%)	145 (100%)	132 (100%)	133 (89%)	548 (97%)	133 (100%)	129 (100%)	133 (99%)	125 (100%)	522 (99.8%)
Grade 1	0	0	0	13 (9%)	13 (2%)	0	0	1 (1%)	0	1 (0.2%)
Grade 2	0	0	0	2 (1%)	2 (0.4%)	0	0	0	0	0
Grade 3	0	0	0	1 (1%)	1 (0.2%)	0	0	0	0	0
Neutropenia	N=138	N=145	N=132	N=149	N=564	N=133	N=129	N=136	N=125	N=523
Grade 0	138 (100%)	144 (99%)	131 (99%)	99 (66%)	512 (91%)	133 (100%)	127 (98%)	135 (99%)	123 (98%)	518 (99%)
Grade 1	0	1 (1%)	1 (0.8%)	35 (23%)	37 (7%)	0	2 (2%)	0	2 (2%)	4 (1%)
Grade 2	0	0	0	15 (10%)	15 (3%)	0	0	1 (1%)	0	1 (0.2%)

Table S18 Priors for analysis of the primary endpoint

	Primary analysis: uninformative	Sensitivity analyses	
		Sceptical	Enthusiastic
Control cure rate	N(2.94, 2.08)	N(2.94, 2.08)	N(2.94, 2.08)
Regimen comparison	N(0, 10000)	N(-0.05, 0.009)	N(0, 0.009)
Strategy comparison	N(0, 10000)	N(-0.1, 0.0036)	N(0, 0.0036)
Other stratification factors*	N(0, 10000)	N(0, 10000)	N(0, 10000)

*HCV genotype, treatment centre.

The SAP specified a control cure rate prior of beta(4.75, 0.25), which had mean 0.95 and variance 0.008, but this was unsuitable for analysis using logistic regression and was changed to N(2.94, 2.08) after discussion with a statistical methodologist.

References

1. WHO. WHO Guidelines for the care and treatment of persons diagnosed with chronic hepatitis C virus infection. Geneva: World Health Organization; 2018.
2. Sterling RK, Lissen E, Clumeck N, et al. Development of a simple noninvasive index to predict significant fibrosis in patients with HIV/HCV coinfection. *Hepatology* 2006; **43**(6): 1317-25.
3. Nitta Y, Kawabe N, Hashimoto S, et al. Liver stiffness measured by transient elastography correlates with fibrosis area in liver biopsy in patients with chronic hepatitis C. *Hepatology research : the official journal of the Japan Society of Hepatology* 2009; **39**(7): 675-84.
4. EASL Clinical Practice Guidelines: Management of hepatitis C virus infection. 2016.
5. Pybus OG, Barnes E, Taggart R, et al. Genetic history of hepatitis C virus in East Asia. *J Virol* 2009; **83**(2): 1071-82.
6. McCabe L, White IR, Chau NVV, et al. The design and statistical aspects of VIETNARMS: a strategic post-licensing trial of multiple oral direct-acting antiviral hepatitis C treatment strategies in Vietnam. *Trials* 2020; **21**(1): 413.
7. Kahan BC, Hindley J, Edwards M, Cro S, Morris TP. The estimands framework: a primer on the ICH E9(R1) addendum. *BMJ* 2024; **384**: e076316.
8. Clements MN, White IR, Copas AJ, et al. Improving clinical trial interpretation with ACCEPT analyses. *NEJM Evid* 2022; **1**(8).
